# Genome-Wide Identification and Characterization of the *GmSnRK2* Family in Soybean

**DOI:** 10.3390/ijms18091834

**Published:** 2017-08-23

**Authors:** Wei Zhao, Yi-Hui Cheng, Chi Zhang, Xin-Jie Shen, Qing-Bo You, Wei Guo, Xiang Li, Xue-Jiao Song, Xin-An Zhou, Yong-Qing Jiao

**Affiliations:** 1 Key laboratory of Biology and Genetic Improvement of Oil Crops, Ministry of Agriculture, Oil Crops Research Institute, Chinese Academy of Agricultural Science, Wuhan 430062, China; zhaowei@caas.cn (W.Z.); chengyihui711@163.com (Y.-H.C.); shenxinjie@caas.cn (X.-J.S.); youqb2006@163.com (Q.-B.Y.); guowei03@caas.cn (W.G.); xiangli@scbg.ac.cn (X.L.); zhouxinan@caas.cn (X.-A.Z.); 2 Beijing Genomics Institute, Wuhan 430075, China; zhangchi@genomics.cn; 3 State Key Laboratory of Crop Biology, Agronomy College, Shandong Agricultural University, Tai’an 271018, China; xjsongsd@163.com

**Keywords:** soybean, sucrose non-fermenting-1 (SNF1)-related protein kinase 2, salinity, abscisic acid, strigolactones

## Abstract

Sucrose non-fermenting-1 (SNF1)-related protein kinase 2s (SnRK2s) that were reported to be involved in the transduction of abscisic acid (ABA) signaling, play important roles in response to biotic and abiotic stresses in plants. Compared to the systemic investigation of *SnRK2s* in *Arabidopsis* *thaliana* and *Oryza sativa*, little is known regarding *SnRK2s* in soybean, which is one of the most important oil and protein crops. In the present study, we performed genome-wide identification and characterization of *GmSnRK2s* in soybean. In summary, 22 *GmSnRK2s* were identified and clustered into four groups. Phylogenetic analysis indicated the expansion of *SnRK2* gene family during the evolution of soybean. Various *cis*-acting elements such as ABA Response Elements (ABREs) were identified and analyzed in the promoter regions of *GmSnRK2s*. The results of RNA sequencing (RNA-Seq) data for different soybean tissues showed that *GmSnRK2s* exhibited spatio-temporally specific expression patterns during soybean growth and development. Certain *GmSnRK2s* could respond to the treatments including salinity, ABA and strigolactones. Our results provide a foundation for the further elucidation of the function of *GmSnRK2* genes in soybean.

## 1. Introduction

Plants are often exposed to various harmful environments, such as salinity, drought, pH variation, temperature and heavy metals. In order to cope with the adverse environmental conditions, plants have developed complex protective mechanisms during evolution [1]. Among them, the phytohormone pathways play important roles in the ability of plants to acclimatize to the stressful environments [2,3]. Abscisic acid (ABA) is one of important phytohormones, which has various physiological functions in mediating plant growth, development and stress responses, such as salt stress and water deficit stress [4,5,6]. Many factors such as Pyrabactin Resistance 1/PYR1-Like/Regulatory Component of ABA Receptor (PYR/PYL/RCAR), Clade A protein phosphatase 2Cs (PP2Cs), and ABA response element-binding factors (ABFs) are involved in the ABA signaling pathway [7]. Particularly, protein phosphorylation and dephosphorylation are essential events during ABA signal transduction [8,9].

SnRK2s belong to the family of sucrose non-fermenting-1 (SNF1)-related protein kinases (SnRKs), which is a class of serine/threonine (Ser/Thr) protein kinases [10,11,12]. The members of *SnRK2s* family have been found in various plant species including *Arabidopsis thaliana* (*AtSnRK2.1–2.10*), *Oryza sativa* (*SAPK1–10*) [11,13], *Malus prunifolia* (*MpSnRK2.1–2.12*) [14], maize (*ZmSnRK2.1–2.11*) [15], wheat (*TaSnRK2.3*, *2.4*, *2.7*, and *2.8*) [16,17,18,19], *Brassica napus* (*BnSnRK2.1–2.14*) [20], *Vitis vinifera* (*VvSnRK2.1–2.6*) [21], Pak-choi (*BcSnRK2.1–2.13*) [22], and cotton (*GhSnRK2.1–2.20*) [23]. Each SnRK2 protein contains three functional domains, including an adenosine triphosphate (ATP) binding domain, a conserved Ser/Thr kinase domain, and a diverse regulatory C-terminal. In general, members of *SnRK2* gene family can be classified into three groups as Group 1, Group 2 and Group 3. The *SnRK2s* of Group 1 are rich of glutamic acid (Glu), while members of Group 2 and Group 3 are rich in aspartic acid (Asp) [24]. Besides, the promoter regions of *SnRK2s* usually harbor *cis-*acting regulatory elements involved in stress responses, such as ABA responsive elements (ABRE), dehydration responsive elements/C-repeat elements (DRE/CRT), and low temperature responsive elements (LTRE) [15].

Recent studies demonstrated that SnRK2s play important roles in ABA signal transduction, which regulates various growth and developmental processes in plants, such as stomatal closure, seed development and dormancy, and flowering time regulation [25,26,27,28,29,30]. In *Arabidopsis*, *SRK2E/SRK2.6* could affect the expression of *RD22* and *RD29B*, which are ABA responsive genes [10]. SRK2D/SnRK2.2, SRK2E/SnRK2.6 and SRK2I/SnRK2.3 are redundant ABA-activated SnRK2s, and play essential roles in controlling seed development and dormancy [27]. The *snrk2.2/2.3/2.6* triple-knockout mutant seedling was insensitive to ABA, produced few seeds, and flowered early [28,29]. Yeast two-hybrid analysis demonstrated that SnRK2.6 could interact with ABI1 (ABA Insensitive 1), which is a PP2C-type phosphatase and plays a critical role in the control of the ABA-induced stomatal closure in *Arabidopsis* [31]. Over-expression of *SRK2C*/*SnRK2.8* could up-regulate stress-induced genes and increase drought tolerance in *Arabidopsis* [32]. In rice, SAPK6 could be activated in rice under dehydration stress, and further phosphorylate OREB1 (*Oryza* ABA-responsive element binding factor 1), a bZIP type transcript factor associated with ABA signaling [33]. Until now, four *SnRK2* genes of *SPK1*, *SPK2*, *SPK3* and *SPK4*, had been reported in soybean, of which, protein kinases SPK1 and SPK2 could be activated by hyperosmotic stress, and *SPK3* and *SPK4* could be induced by high salinity or dehydration [34,35].

Soybean, a leading oil and protein crop around the world, is an ancient tetraploid with a partially diploidized tetraploid [36]. Although several *GmSnRK2s* have been characterized, comprehensive analysis of *GmSnRK2s* at the genome-wide level in soybean is still needed. In this study, we identified and characterized the *GmSnRK2* gene family at the genome-wide level in soybean. The *cis*-acting regulatory elements in the promoter regions of *GmSnRK2s* were also investigated. Based on the publicly available transcriptome data and quantitative real-time polymerase chain reaction (qRT-PCR) results, the expression patterns of *GmSnRK2s* were profiled at different tissues as well as different treatments including salinity, ABA and strigolactones (SLs). Our study will extend understanding of the *GmSnRK2* gene family in soybean, and provide useful information for scientists to analyze the functions of *GmSnRK2* in the future.

## 2. Results

### 2.1. Identification and Properties of SnRK2 Genes in Soybean

To investigate *SnRK2* gene family in soybean, the peptides of SnRK2s of *Arabidopsis thaliana* and rice were used as queries to screen the soybean genome in silico (Table S1). Combined with the functional domains predicted by InterProScan (version 5.25-64.0, Hinxton, Cambridge, UK), we eventually determined 22 putative *GmSnRK2* genes in the soybean genome (Table 1). These *GmSnRK2* genes were distributed in 12 different chromosomes, of which, chromosome 5 (Chr05) contained the highest count of *GmSnRK2* genes including *GmSnRK2.6*, *GmSnRK2.7*, *GmSnRK2.8*, and *GmSnRK2.9*. The previously reported four *GmSnRK2* genes, *SPK1*, *SPK2*, *SPK3,* and *SPK4*, were named as *GmSnRK2.2*, *GmSnRK2.16*, *GmSnRK2.15* and *GmSnRK2.5* in this study, respectively [34,35]. The transcript length of *GmSnRK2s* ranged from 1.3 to 2.2 kb, but the length of GmSnRK2 proteins ranged from 309 to 364 aa (amino acid). Generally, the *SnRK2* gene family has two conserved signatures: An adenosine triphosphate (ATP)-binding region signature, and a Serine/Threonine protein kinase active-site signature [13]. In our results, all the SnRK2s of soybean contained ATP binding sites (IPR017441) and Serine/threonine-protein kinase active sites (IPR008271) at the N-terminal (Table 1).

To explore the physical and chemical properties of these soybean SnRK2 proteins, the peptides of GmSnRK2s were profiled by ProtParam (available online: http://web.expasy.org/protparam/). The molecular weight of GmSnRK2s ranged from 35.37 to 41.49 kDa. The isoelectric point varied from 4.68 to 6.48, which revealed that GmSnRK2 proteins were acidic. The Grand Average of Hydropathy (GRAVY) values of GmSnRK2s were all negative, indicating that GmSnRK2s were hydrophilic and probably located in cytosol (Table 1).

**Figure 1:**
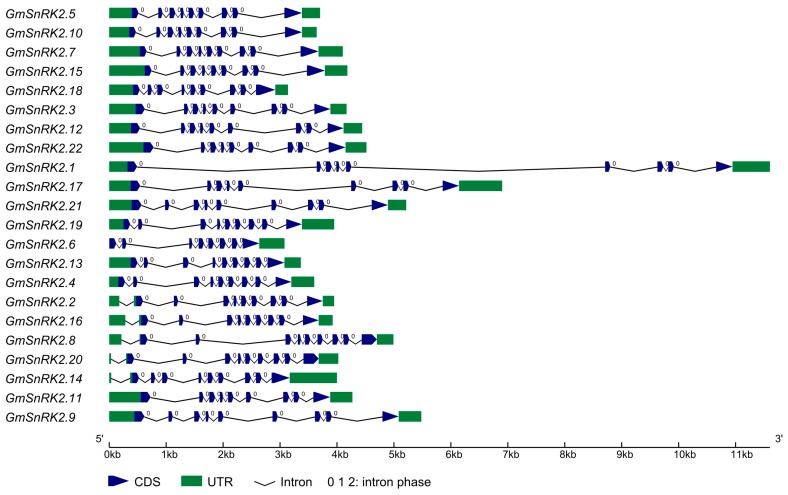
Gene structure of *GmSnRK2* genes. Introns are represented by black lines. Exons are represented by blue wedges. 5′ and 3′ untranslated regions (UTRs) are represented by green boxes. The intron phase is labeled above each intron. The scale of gene length is given at the bottom. CDS: Coding sequence.

Based on the inferred gene structure, *GmSnRK2s* exhibited similar exon-intron organizations and highly conserved intron phasing (Figure 1). The coding sequences of *GmSnRK2s* were disrupted by eight introns except for *GmSnRK2.6*, and the intron sizes varied greatly, ranging from 78 to 4640 bp (base pair).

### 2.2. Phylogenetic and Motif Analysis of SnRK2 Genes in Soybean

**Figure 2:**
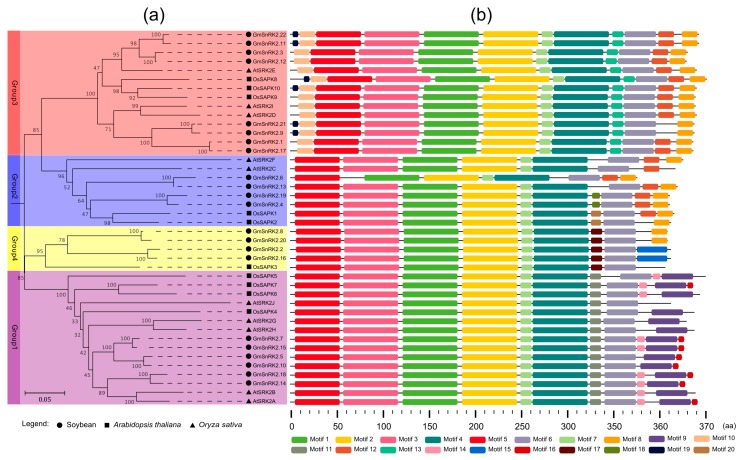
Phylogenetic tree and protein motif analysis of GmSnRK2s. (**a**) Phylogenetic tree of SnRK2s of *Arabidopsis thaliana*, *Oryza sativa* and soybean was constructed by the Neighbor-Joining (NJ) method using MEGA5 software (version 5.1 β, Hachioji, Tokyo, Japan). Group 1, Group 2, Group 3 and Group 4 are marked as purple, blue, red and yellow, respectively. Soybean, *Arabidopsis thaliana* and *Oryza sativa SnRK2s* are represented by black circle, square and triangle, respectively; (**b**) protein motifs of GmSnRK2s, AtSnRK2s and OsSnRK2s profiled by MEME (version 4.12.0, Seattle, WA, USA). The 20 conserved motifs are represented by colored boxes at the bottom. The scale of protein length is given below the schematic diagram.

To investigate the evolutionary relationships of the *SnRK2* genes among *Arabidopsis thaliana*, rice and soybean, a neighbor-joining phylogenetic tree was constructed using the protein sequences of AtSnRK2s, OsSnRK2s and GmSnRK2s. These SnRK2s were clustered into four subgroups named as Group 1, Group 2, Group 3 and Group 4 (Figure 2a). Consistent with previous report, the 10 *SnRK2s* of *Arabidopsis thaliana* were distributed within three groups: Group 1, Group 2 and Group 3 [37]. The phylogenetic tree showed that Group 3 was the biggest group, with eight members. The next biggest group was Group 1 that had six *GmSnRK2s*. Group 2 had four *GmSnRK2s*. Group 4 was the smallest clade constituted by four *GmSnRK2s* and *OsSAPK3*. Compared with *Arabidopsis thaliana* and rice, soybean exhibited an expansion of *SnRK2* gene family in each group (Figure 2a). Most of the *GmSnRK2s* clustered together in pairs in the phylogenetic tree, such as the clade of *GmSnRK2.1*, *GmSnRK2.9*, *GmSnRK2.17* and *GmSnRK2.21*, the clade of *GmSnRK2.2*, *GmSnRK2.8*, *GmSnRK2.16* and *GmSnRK2.20*, the clade of *GmSnRK2.3*, *GmSnRK2.11*, *GmSnRK2.12* and *GmSnRK2.22*, and the clade of *GmSnRK2.5*, *GmSnRK2.7*, *GmSnRK2.10* and *GmSnRK2.15*.

The conserved motifs of *AtSnRK2s*, *OsSnRK2s* and *GmSnRK2s* were identified using MEME (version 4.12.0, Seattle, WA, USA), and a total of 20 different motifs were found (Figure 2b). Six motifs (motifs 1–5 and motif 7) constituted the highly conserved N-terminal and were shared in almost all of the *SnRK2s*, whereas in *GmSnRK2.6* motif 3 was lacking. However, in the C-terminal regions of SnRK2s, the motifs were diverse in each group. For example, motif 10 and motif 13 were specific to Group 3, while motif 9, motif 11, motif 14, and motif 16 only existed in Group 1. Similarly, motif 18 and motif 20 were specific to Group 2, while motif 15 and motif 17 were only found in Group 4. In addition, members of Group 2 and Group 3 contained motif 8 and motif 12 in the *C*-terminal. Most of the *GmSnRK2* gene pairs contained the same motifs, such as *GmSnRK2.1* and *GmSnRK2.17*, *GmSnRK2.2* and *GmSnRK2.16*, *GmSnRK2.3* and *GmSnRK2.12*, *GmSnRK2.4* and *GmSnRK2.19*, *GmSnRK2.5* and *GmSnRK2.10*, *GmSnRK2.7* and *GmSnRK2.15*, *GmSnRK2.8* and *GmSnRK2.20*, *GmSnRK2.9* and *GmSnRK2.21*, *GmSnRK2.11* and *GmSnRK2.22*, and *GmSnRK2.14* and *GmSnRK2.18*.

The sequence homology of GmSnRK2 proteins was determined through multiple sequence alignment. GmSnRK2s exhibited an average of 66.7% amino acid sequence identity (Figure S1). Their N-terminals were mainly constituted by ATP binding domain and highly conserved serine/threonine protein kinases domain (Figure S1). Motif 5 represented the ATP binding domain, and motif 2 represented the conserved serine/threonine protein kinases domain (Figure 2). Similar to SnRK2s in *Arabidopsis thaliana* and *rice*, the GmSnRK2s of Group 1 showed an enrichment of Glu (E) at the C-terminal, while the members of Group 2 and Group 3 were Asp (D)-rich in soybean. However, the GmSnRK2s of Group 4 had no such D/E-rich pattern (Figure S1). Notably, GmSnRK2.6, the shortest SnRK2 of soybean, lacked 34 amino acid residues at the conserved *N*-terminal domain in comparison with other GmSnRK2s.

### 2.3. Cis-Acting Regulatory Elements in the Promoters of GmSnRK2 Genes

The genomic DNA sequences from the transcription initiation site (+1) to the 2 kb upstream of the *GmSnRK2* genes were extracted for *cis*-acting elements profiling using PlantCARE. The results obtained from PlantCARE server showed that various *cis*-acting regulatory elements existed within promoter regions of *GmSnRK2s* (Table 2 and Table S3). For example, nine *GmSnRK2s*, including *GmSnRK2.1*, *GmSnRK2.9*, *GmSnRK2.12*, *GmSnRK2.17* and *GmSnRK2.21* of Group 3, *GmSnRK2.18* of Group 1, *GmSnRK2.4*, and *GmSnRK2.13* and *GmSnRK2.19* of Group 2, had one or more ABREs. However, there were no ABREs found in the promoter regions of all the *GmSnRK2s* of Group 4 (Table 2). In addition, some other phytohormone response elements except for ABREs, such as AuxRR-core, P-box, TCA-element, TGACG-motif and ethylene response element (ERE), were also present in the promoter regions of *GmSnRK2s*, which suggested that the expression of *GmSnRK2s* might be also induced by auxin, salicylic acid, methyl jasmonic acid, gibberellin and ethylene (Table 2).

Notably, the *cis*-acting elements involved in response to biotic and abiotic stresses, including fungus, drought, heat and low-temperature, were also found within the upstream regions of *GmSnRK2* genes, indicating that *GmSnRK2s* might be also induced by these biotic and abiotic stresses (Table S3). Moreover, there were 14 *GmSnRK2s* containing several *cis*-acting regulatory elements involved in circadian control, suggesting that the expression of these *GmSnRK2s* might be associated with circadian control (Table S3).

### 2.4. Expression Analysis of GmSnRK2 Genes during Growth and Development of Soybean

**Figure 3:**
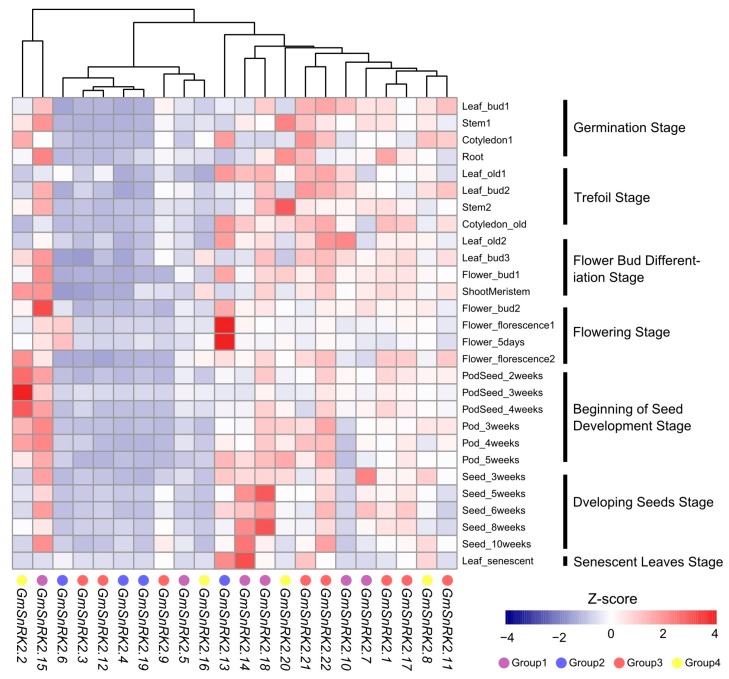
Expression analysis of *GmSnRK2s* during growth and development of different tissues of soybean. The RNA sequencing (RNA-Seq) dataset of 28 tissues of soybean collected from different development stages were obtained from the National Center for Biotechnology Information (accession number: SRP038111) to generate heatmap. The color scale indicating Z-score value is given below. Group 1, Group 2, Group 3 and Group 4 are marked as purple, blue, red and yellow circles, respectively. The different stages are marked by vertical black lines.

To examine the expression patterns of the *GmSnRK2s* during growth and development, we re-analyzed of the publicly available RNA-Seq dataset (SRP038111), which was obtained from different tissues at different developmental stages of soybean. *GmSnRK2.2* of Group 4 was highly expressed in dividing and meristem tissues of young seedlings, stems and pods, but lowly expressed in seeds. *GmSnRK2.16* of Group 4 was preferentially expressed in shoot meristem and leaf buds at the flower bud differentiation stage. *GmSnRK2.15* and *GmSnRK2.22* showed constitutive expression at high levels in all the examined tissues except for the senescent leaves. In contract, *GmSnRK2.3*, *GmSnRK2.4*, *GmSnRK2.5*, *GmSnRK2.9*, *GmSnRK2.12* and *GmSnRK2.19* showed relatively low expression levels in all the tissues. *GmSnRK2.6* and *GmSnRK2.13* were paralogous gene pairs, and both of them exhibited specifically higher expression in florescence flowers than in other tissues. Notably, during the development of seed, the expression of *GmSnRK2.14* and *GmSnRK2.15* of Group 1 were increased as the seed developed. *GmSnRK2.7* of Group 1 exhibited relatively high expression level in the early stages of seed development, and *GmSnRK2.18* of Group 1 showed relatively high expression level in the mid stages of seed development (Figure 3). Overall, the *GmSnRK2* genes displayed diverse spatio-temporal expression patterns in soybean.

### 2.5. Differential Expression of GmSnRK2s in Response to Salt Stress, Abscisic Acid and Strigolactones

The members of *SnRK2* gene family play important roles in response to various environmental stresses, such as hyperosmotic stress, high salinity and dehydration [24,38]. Besides, *SnRK2s* also play critical roles in regulation of phytohormone pathways, especially ABA signal transduction [25,39,40,41]. To explore the expression patterns of *GmSnRK2* genes in response to biotic stresses and phytohormones, the expression levels of *GmSnRK2s* were evaluated by qRT-PCR under NaCl, ABA, and a synthetic analog of strigolactones (*rac*-GR24) treatments in soybean.

**Figure 4:**
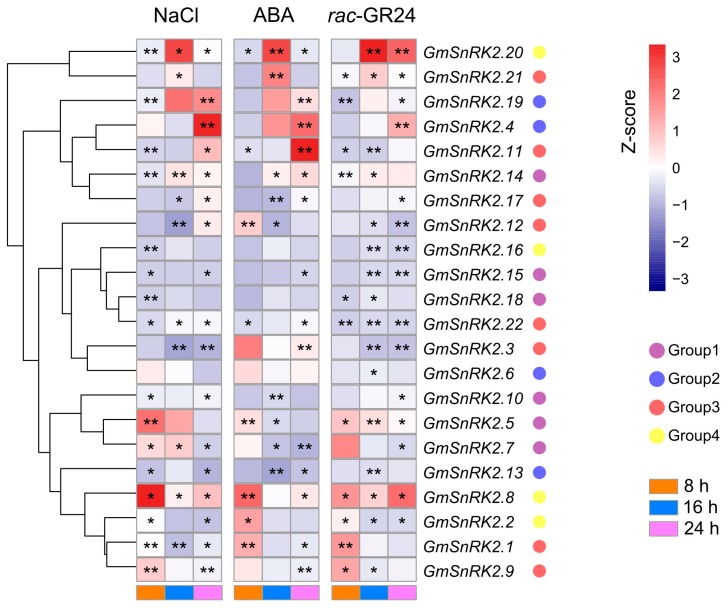
Expression analysis of the *GmSnRK2s* under salt, abscisic acid (ABA) and strigolactones (SLs) treatments. Heatmaps showed the expression patterns in quantitative real time polymerase chain reaction (qRT-PCR) experiments for the 22 *GmSnRK2* genes under the treatments of NaCl, ABA and *rac*-GR24. The transcript levels of the *GmSnRK2s* were normalized against *GmSKIP* transcript levels using 2^−ΔΔ*C*t^. The clustered heatmaps was generated in R using the pheatmap function package. The color scale indicating Z-score value, the Group names of *GmSnRK2s* and time points are shown at the right. Statistically significant differences (Student’s *t*-test) are indicated as follows: * *p* < 0.05, ** *p* < 0.01.

To elucidate the roles of *GmSnRK2s* under high salinity stress condition, the expression levels of 22 *GmSnRK2* genes were examined in the leaves of seedlings of soybean under NaCl treatment at four time points: 0, 8, 16 and 24 h. Of the 22 *GmSnRK2* genes, *GmSnRK2.7*, *GmSnRK2.8*, *GmSnRK2.14*, *GmSnRK2.20* and *GmSnRK2.22* were significantly up-regulated at three time points (8, 16 and 24 h) under NaCl stress compared to the control (*p*-value ≤ 0.05, Figure 4 and Figure S2). At 8 h after treatment, except for *GmSnRK2.12* and *GmSnRK2.13*, all the other *GmSnRK2* genes showed increased expression, among which *GmSnRK2.5* and *GmSnRK2.8* were significantly up-regulated by approximately 13- and 18-fold, respectively (Figure S2). The expression levels of *GmSnRK2.2* (*SPK1*) and *GmSnRK2.5* (*SPK4*) were up-regulated significantly at 8 h, and then were down-regulated to the basal level at 24 h, whereas the expression levels of *GmSnRK2.16* (*SPK2*) and *GmSnRK2.15* (*SPK3*) were increased slightly at each time point under salt stress, which suggested that the induction kinetics of the four *GmSnRK2s* under high salinity were different. The expression levels of *GmSnRK2.1* and *GmSnRK2.9* of Group 3 were significantly up-regulated at 8 h, and then down-regulated at 16 h after salt treatment (Figure 4a). The expression level of *GmSnRK2.4* was increased from 1.1-fold at 8 h to 8.5-fold at 24 h after treatment compared to the control (Figure S2). In addition, *GmSnRK2.6* expression showed no difference at each time point (Figure 4).

To expand our understanding of the physiological functions of *GmSnRK2* genes in response to phytohormones, we examined their expression levels under exogenous ABA and SLs treatments using qRT-PCR. Following the ABA treatment, all *GmSnRK2s*, except for *GmSnRK2.6*, *GmSnRK2.16* and *GmSnRK2.18*, showed differential expression at different time points after ABA treatment (*p*-value ≤ 0.05, Figure 4 and Figure S3). At 8 h after treatment, the expression levels of *GmSnRK2.1*, *GmSnRK2.2*, *GmSnRK2.5*, *GmSnRK2.8* and *GmSnRK2.12* were significantly up-regulated, while the expression levels of *GmSnRK2.11*, *GmSnRK2.12* and *GmSnRK2.20* were significantly down-regulated (Figure 4). At 16 h after treatment, *GmSnRK2.7* and *GmSnRK2.12* were down-regulated from 2.9-fold at 8 h to 0.5-fold at 16 h and from 3.9-fold at 8 h to 0.4-fold at 16 h, respectively (Figure S3). Besides, the expression levels of *GmSnRK2.10*, *GmSnRK2.13* and *GmSnRK2.17* were also significantly reduced nearly by half compared with the control (Figure 4 and Figure S3). The expression of *GmSnRK2.20* and *GmSnRK2.21* were significantly up-regulated at 16 h compared to the control after ABA treatment (Figure 4). At 24 h after treatment, *GmSnRK2.4* and *GmSnRK2.11* expression levels were significantly up-regulated from 1.5-fold at 8 h to 7.1-fold at 24 h and from 1.7-fold at 8 h to 9.5-fold at 24 h, respectively (Figure S3). The expression levels of *GmSnRK2.3* and *GmSnRK2.19* were slightly increased, while the expression of *GmSnRK2.7* and *GmSnRK2.9* were significantly down-regulated at 24 h compared to the control after ABA treatment (Figure 4).

To investigate the response of *GmSnRK2s* to SLs, a synthetic analog of strigolactones (*rac*-GR24) was applied to treat soybean seedlings. The results of qRT-PCR showed that SLs treatment caused a significant induction of the expression of nine *GmSnRK2* genes including *GmSnRK2.1*, *GmSnRK2.2*, *GmSnRK2.4*, *GmSnRK2.5*, *GmSnRK2.7*, *GmSnRK2.8*, *GmSnRK2.9*, *GmSnRK2.20*, and *GmSnRK2.21* (fold change ≥ 2 and *p*-value ≤ 0.05, Figure 4 and Figure S4). The expression levels of *GmSnRK2.5* and *GmSnRK2.8* were significantly up-regulated, whereas the expression of *GmSnRK2.22* was down-regulated at each examined time point compared to the control after treatment (Figure 4). Members of Group 3, such as *GmSnRK2.3*, *GmSnRK2.11*, *GmSnRK2.12* and *GmSnRK2.22* were significantly down-regulated after SLs treatment at different time points. Both *GmSnRK2.3* and *GmSnRK2.12* were down-regulated from 1.2-fold at 8 h to 0.1-fold at 24 h, whereas *GmSnRK2.19* of Group 2 was up-regulated from 0.3-fold at 8 h to 1.4-fold at 24 h after treatment compared to the control (Figure 4 and Figure S4). Members of Group 4 exhibited diverse expression patterns. For example, the paralogous gene pairs of *GmSnRK2.8* and *GmSnRK2.20* showed significantly up-regulated expression, while the paralogous gene pairs of *GmSnRK2.2* and *GmSnRK2.16* showed significantly down-regulated expression at 16 and 24 h compared to the control after treatment (Figure 4).

The expression of *GmSnRK2s* of Group 1, except for *GmSnRK2.18*, exhibited significant changes at different time points compared to the control after NaCl, ABA and SLs treatments. Notably, under NaCl, ABA and SLs treatments, *GmSnRK2.5* (*SPK4*) showed similar expression patterns at different conditions, as well as *GmSnRK2.14* (Figure 4). The expression of *GmSnRK2.4* of Group 2 were significantly up-regulated at 24 h after NaCl, ABA and SLs treatments, while *GmSnRK2.6* of Group 2 only showed differential expression at 16 h after SLs treatment (Figure 4). The expression of *GmSnRK2.3* of Group 3 was down-regulated after NaCl and SLs treatments, while up-regulated after ABA treatment. Besides, the expression of *GmSnRK2.12* of Group 3 was significantly down-regulated, while the expression of *GmSnRK2.21* of Group 3 was significantly up-regulated at 16 h compared to the control after NaCl, ABA and SLs treatments (Figure 4). The expression levels of *GmSnRK2.2* and *GmSnRK2.8* were significantly up-regulated at 8 hr compared to the control after NaCl, ABA and SLs treatments (Figure 4).

*GmSnRK2.4* and *GmSnRK2.19* of Group 2 shared similar expression patterns, and both showed up-regulated expression at 24 h after ABA and NaCl treatments (Figure 4). *GmSnRK2.4* and *GmSnRK2.19* harbored one ABRE and one GARE-motif (gibberellin response element-motif) in their promoters (Table 2). Similarly, *GmSnRK2.16* of Group 4 and *GmSnRK2.22* of Group 3 exhibited down-regulated expression at 8 h after NaCl treatment, and both were also down-regulated at 16 and 24 h after SLs treatment compared to the control (Figure 4). Meanwhile, *GmSnRK2.22* showed significantly differential expression, whereas the expression of *GmSnRK2.16* had no changes after ABA treatment compare to the control (Figure 4). Both *GmSnRK2.16* and *GmSnRK2.22* had no ABREs, and contained some other phytohormone response elements in their promoters, including TGACG-motif, CGTCA-motif and TCA-element (Table 2). However, *GmSnRK2.22* had one more TCA-element and TC-rich repeats element than *GmSnRK2.16* (Table 2 and Table S3). By contrast, *GmSnRK2.14* and *GmSnRK2.18* exhibited different expression patterns under ABA and NaCl treatments. The expression of *GmSnRK2.18* had no changes, while the expression of *GmSnRK2.14* was significantly up-regulated at 16 and 24 h after ABA and NaCl treatments. *GmSnRK2.18* contained one ABRE, whereas *GmSnRK2.14* had no ABREs in the promoters; in addition, *GmSnRK2.14* had one TATC-box (Table 2).

### 2.6. Co-Regulatory Networks of GmSnRK2s

**Figure 5:**
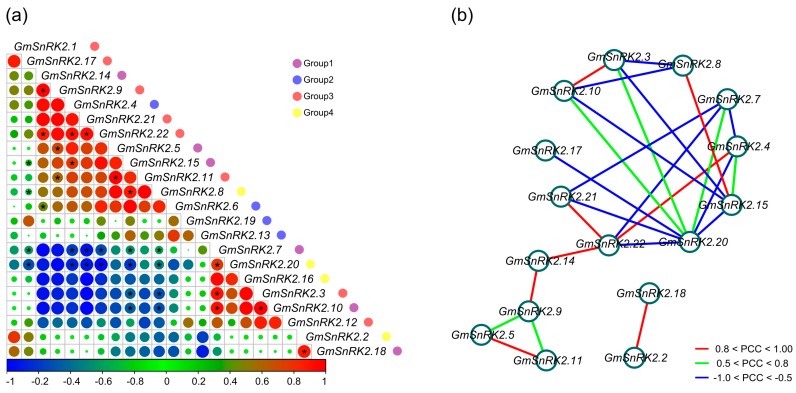
Correlations and co-regulatory networks of *GmSnRK2* genes during seed development. (**a**) Correlation analysis of *GmSnRK2* genes during seed development were performed based on the Pearson correlation coefficients (PCCs) of gene pairs calculated using R package program (version 3.2.2; available online: https://cran.r-project.org/web/packages/pheatmap/). Correlations are indicated by the size and color of circles. Below bar represents the correlation values of Pearson correlation coefficients (PCCs). Red and blue colors indicate positive correlation and negative correlation, respectively. Gene names are listed at the right. Group 1, Group 2, Group 3 and Group 4 are marked as purple, blue, red, and yellow circles, respectively. Black star represents the correlation with *p*-value ≤ 0.05; (**b**) co-regulatory network of *GmSnRK2s* during seed development was illustrated by Cytoscape (version 3.1, Seattle, WA, USA). The significant PCCs of gene pairs (*p*-value ≤ 0.05) are included, and the different correlation levels of gene pairs are marked by edge lines with different colors, as showed below the co-regulatory networks.

By using the publicly available RNA-Seq dataset (SRP038111), we investigated the correlations of the 22 *GmSnRK2s* during seed development of soybean. We calculated the Pearson correlation coefficients (PCCs) of the expression levels of *GmSnRK2s*, and constructed co-regulatory networks. The expression profiles of *GmSnRK2.14* of Group 1, *GmSnRK2.4* of Group 2, and *GmSnRK2.9*, *GmSnRK2.21* and *GmSnRK2.22* of Group 3 exhibited positive correlations between one another. *GmSnRK2.2* of Group 4 and *GmSnRK2.18* of Group 1 showed positive correlation, and both showed negative correlations with *GmSnRK2.13* of Group 2. Similarly, *GmSnRK2.3* of Group 3, *GmSnRK2.10* of Group 1, and *GmSnRK2.16* of Group 4 exhibited positive correlations between each other, and all showed negative correlations with *GmSnRK2.5*, *GmSnRK2.14* and *GmSnRK2.15* of Group 1, *GmSnRK2.4* and *GmSnRK2.6* of Group 2, and *GmSnRK2.9*, *GmSnRK2.11*, *GmSnRK2.21* and *GmSnRK2.22* of Group 3 (Figure 5a). All the significant PCCs (*p*-value ≤ 0.05 and PCC > 0.5) of *GmSnRK2*s were extracted and used to construct seed development co-regulatory networks delineated by Cytoscape (version 3.1, Seattle, WA, USA) (Figure 5b). A total of 26 edges and 16 nodes were in the co-regulatory networks. There were 14 *GmSnRK2* gene pairs showing positive correlations, of which eight pairs exhibited strongly positive correlations (*p*-value ≤ 0.05 and PCC > 0.8), including *GmSnRK2.2* and *GmSnRK2.18*, *GmSnRK2.3* and *GmSnRK2.10*, *GmSnRK2.4* and *GmSnRK2.22*, *GmSnRK2.5* and *GmSnRK2.11*, *GmSnRK2.8* and *GmSnRK2.15*, *GmSnRK2.9* and *GmSnRK2.14*, *GmSnRK2.14* and *GmSnRK2.22*, and *GmSnRK2.21* and *GmSnRK2.22*. Meanwhile, twelve *GmSnRK2* gene pairs showed negative correlations (*p*-value ≤ 0.05 and PCC < −0.5) (Figure 5b).

**Figure 6:**
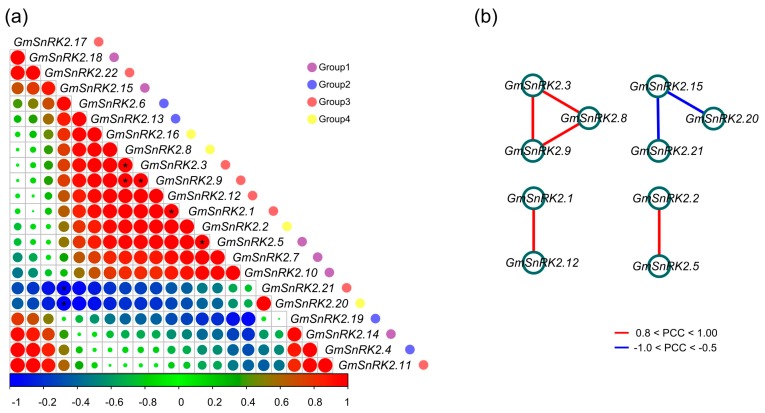
Correlations and co-regulatory networks of *GmSnRK2* genes under ABA treatment. (**a**) Correlation analysis of *GmSnRK2* genes under ABA treatments were performed based on the PCCs of gene pairs calculated using R package program. Correlations were indicated by the size and color of circles. Below bar represented the correlation values of PCCs. Red and blue indicate positive correlation and negative correlation, respectively. Gene name was listed at the right. Group 1, Group 2, Group 3 and Group 4 were marked as purple, blue, red and yellow circles, respectively. Black star represented the correlation with *p*-value ≤ 0.05; (**b**) co-regulatory network of *GmSnRK2s* under ABA treatment was illustrated by Cytoscape. The significant PCCs of gene pairs (*p*-value ≤ 0.05) were included, and the different correlation levels of gene pairs were marked by edge line with different colors as showed below the co-regulatory networks.

Based on the qRT-PCR results, we also separately explored the correlations of *GmSnRK2s* in response to salt stress, ABA and SLs. Under ABA treatment, positive correlations were observed between *GmSnRK2s*, such as *GmSnRK2.5*, *GmSnRK2.7* and *GmSnRK2.15* of Group 1, *GmSnRK2.6* and *GmSnRK2.13* of Group 2, *GmSnRK2.1*, *GmSnRK2.3*, *GmSnRK2.9* and *GmSnRK2.12* of Group 3, and *GmSnRK2.2*, *GmSnRK2.8* and *GmSnRK2.16* of Group 4 (Figure 6a). Among them, *GmSnRK2.1*, *GmSnRK2.3*, *GmSnRK2.5*, *GmSnRK2.6*, *GmSnRK2.7*, *GmSnRK2.8*, *GmSnRK2.9* and *GmSnRK2.16* also showed positive correlations between each other under NaCl and SLs treatments (Figure S5). In addition, *GmSnRK2.19* of Group 2, *GmSnRK2.20* of Group 4, and *GmSnRK2.21* of Group 3 showed negative correlation with most of the other *GmSnRK2s* under ABA treatment (Figure 6a). Particularly, *GmSnRK2.20* only showed strongly positive correlation with *GmSnRK2.21* of Group 3, *GmSnRK2.13* of Group 2, and *GmSnRK2.19* of Group 2 under ABA, NaCl and SLs treatments, respectively (Figure 6a and Figure S5).

The ABA-related, SLs-related and salinity-related co-regulatory networks of *GmSnRK2*s were also constructed separately. The ABA-related co-regulatory network of *GmSnRK2s* was constituted with seven edges and 10 nodes (Figure 6b). Five *GmSnRK2* gene pairs showed significantly positive correlations (*p*-value ≤ 0.05 and PCC > 0.8), including *GmSnRK2.1* and *GmSnRK2.12*, *GmSnRK2.2* and *GmSnRK2.5*, *GmSnRK2.3* and *GmSnRK2.8*, *GmSnRK2.3* and *GmSnRK2.9*, *GmSnRK2.8* and *GmSnRK2.9*. In addition, two *GmSnRK2* gene pairs showed negative correlations (*p*-value ≤ 0.05 and PCC < −0.5) under ABA treatment (Figure 6b). The positive correlations among *GmSnRK2.3*, *GmSnRK2.8* and *GmSnRK2.9* also existed in the NaCl-related co-regulatory network of *GmSnRK2s* (Figure S5). Similarly, the positive correlation between *GmSnRK2.11* and *GmSnRK2.17* was present in both NaCl-related and SLs-related co-regulatory networks of *GmSnRK2s* (Figure S5). Notably, the significant correlations under NaCl, ABA and SLs treatments existed not only between *GmSnRK2s* of the same “Group”, such as *GmSnRK2.3* of Group 3 and *GmSnRK2.9* of Group 3 in the ABA-related and NaCl-related co-regulatory networks, and *GmSnRK2.5* of Group 1 and *GmSnRK2.7* of Group 1 in the SLs-related co-regulatory network, but also between members of the different “Group”, such as *GmSnRK2.2* of Group 4 and *GmSnRK2.5* of Group 1 in the ABA-related co-regulatory network, *GmSnRK2.4* of Group 2 and *GmSnRK2.22* of Group 3 in the NaCl-related co-regulatory network, and *GmSnRK2.10* of Group 1 and *GmSnRK2.11* of Group 3 in the SLs-related co-regulatory network (Figure 6b and Figure S5). However, among all the correlations in the ABA-related, salt-related and SLs-related co-regulatory networks, no significant correlations were observed between the paralogous gene pairs of *GmSnRK2s* (Figure 6b and Figure S5). In the co-regulatory networks, the *GmSnRK2* genes with significant correlation contained common *cis*-active elements in their promoters. For example, *GmSnRK2.3* and *GmSnRK2.9* contained ERE, GARE-motif, MYB binding site involved in drought-inducibility (MBS) and TC-rich repeats elements in their promoters. *GmSnRK2.1* and *GmSnRK2.12* had ABRE, AuxRR-core, TCA-element and TC-rich repeats elements in their promoter regions. Additionally, *GmSnRK2.2*, *GmSnRK2.5* and *GmSnRK2.7* had MeJA response and TC-rich repeats elements, while *GmSnRK2.10*, *GmSnRK2.11* and *GmSnRK2.17* harbored MBS, heat stress responsiveness (HSE) and TC-rich repeats element in the promoters (Table 2 and Table S3).

## 3. Discussion

While being exposed to the harmful environments, plants could acclimate themselves to the stresses through various morphological, physiological, and molecular responses [3]. Among these processes, phosphorylation by stress-inducible protein kinases plays important roles for plants to sense and respond to environmental stresses [9]. *SnRK2s*, a subfamily of Ser/Thr protein kinases family, had critical functions in responses to hyperosmotic stress, high salinity and dehydration [24,38]. Besides, *SnRK2s* also play important roles in ABA signal transduction [25,39,41]. Here, we performed a comprehensive identification and characterization of soybean *SnRK2* genes (*GmSnRK2s*). Compared to the number of *SnRK2s* found in other diploid plant species, such as 10 in *Arabidopsis thaliana* [11], 10 in *Oryza sativa* [13], 12 in *Malus prunifolia* [14] and 11 in maize [15], soybean contained much more *SnRK2* genes, including 22 *SnRK2*s over the 12 chromosomes.

The exon-intron organizations of *GmSnRK2s* exhibited high similarity with *Arabidopsis thaliana* and rice [13]. The sizes of introns of *GmSnRK2s* varied a lot, but their intron phasing is highly conserved. Except for *GmSnRK2.6*, the coding sequences of *GmSnRK2s* were separated by eight introns, and most of *GmSnRK2s* contained nine exons. The similar distribution of eight introns and nine exons was also found in other species, such as *Arabidopsis thaliana*, rice, cotton and maize, indicating the high evolutionary conservation of gene structure of *SnRK2s* in higher plants [11,12,13,14,15].

Phylogenetic analysis revealed that *SnRK2*s of soybean were primarily classified into four phylogenetic groups as Group 1, Group 2, Group 3 and Group 4. Previous study demonstrated that *SnRK2s* of *Arabidopsis thaliana* and cotton were clustered into three groups [13,23,37]. *SnRK2s* of Group 1 and Group 2 were only activated under hyperosmotic stress [11], while members of Group 3 could be response to both ABA and hyperosmotic signal [31,37]. Unlike Group 1, Group 2 and Group 3, *GmSnRK2s* of the new subgroup of Group 4 with four *GmSnRK2s* and *OsSAPK3* showed no enrichment for Glu(E) or Asp(D) in the C-terminal. The member of Group 4, *OsSAPK3*, was reported to be up-regulated by ABA treatment, and down-regulated by mannitol treatment in the roots [11]. Similarly, the expression levels of several members of Group 4 in soybean, such as *GmSnRK2.2*, *GmSnRK2.8* and *GmSnRK2.22*, were significantly up-regulated after ABA treatment. In addition, *OsSAPK3* could be autophosphorylated in a Ca^2+^-dependent manner and its expression was also up-regulated by *Xanthomonas oryzae* pv. *oryzicola* infection [42,43]. These indicated that the *GmSnRK2s* of Group 4 might be involved in response to both abiotic and biotic stresses. In all the four groups, *SnRK2s* of soybean showed expansion, which might be the result from two whole genome duplication events in the long-term evolution of soybean [36].

Previous report demonstrated that multiple ABREs or an ABRE coupled with other elements such as DRE/CRT was required for the expression of ABA-responsive genes [44]. Various *cis*-acting regulatory elements were found in the promoter regions of *GmSnRK2* genes. Among those are the regulatory elements known to be involved in ABA response, defense and stress responsiveness, suggesting their roles under various biotic and abiotic stimuli. ABA plays important roles in a diverse range of biological processes including development and response to drought, salinity, cold and biotic stress [4,5]. The *SnRK2s* family plays a crucial role in plant response to abiotic stresses and ABA-dependent plant development [25,26,27,28,29,30]. In *Arabidopsis*, *SRK2C*, *SRK2D*, *SRK2E*, *SRK2F*, and *SRK2I* could be activated by ABA [8,28,29]. In rice, three members of *SnRK2* genes (*OsSAPK8*, *OsSAPK9* and *OsSAPK10*) were activated by ABA [11]. In this study, we demonstrated that the expression of 19 *GmSnRK2* genes could be induced by exogenous ABA treatment in soybean. Interestingly, among three *GmSnRK2* genes that did not respond to ABA treatment, *GmSnRK2.6* and *GmSnRK2.16* had no ABREs, and *GmSnRK2.18* had only one ABRE in their promoter regions. The expression of *GmSnRK2.5* (*SPK4*) was reported to be not affected by exogenous ABA previously [34]. However, our data exhibited that the expression level of *GmSnRK2.5* was significantly increased at 8 h after ABA treatment, and then declined to the basal level at 16 and 24 h. This conflicting result might be caused by different concentration of exogenous ABA (100 μM for this study; 200 μM for previous study) and time points (0, 8, 16 and 24 h for this study; 0, 1, 3, 6, 12 and 24 h for previous study) that were used for treatment and detection. Additionally, we applied salt stress on soybean seedlings to explore the expression changes of *GmSnRK2s*. The expressions of most of *GmSnRK2s* were induced by salt stress. *GmSnRK2.6* did not respond to NaCl treatment, while *GmSnRK2.16* and *GmSnRK2.18* showed significantly differential expression after NaCl treatment. *GmSnRK2.16* and *GmSnRK2.18* contained two MeJA response elements in their promoters compared to *GmSnRK2.6*, which had no MeJA response element in the promoter region. Members of *GmSnRK2* gene family showed different and temporally dynamic expression patterns after NaCl and ABA treatments. The diverse expression patterns of *GmSnRK2s* under stresses might be attributed to different *cis*-acting elements in their promoters, which is worthy of being studied further. Although some SnRK2s were major regulators of ABA signal transduction, their transcript or protein levels might not be affected by ABA application, because the activities of SnRK2s were transiently regulated via reversible phosphorylation in plant cells [11,12]. Thus, to confirm the involvement of ABA pathway for the *GmSnRK2s*, further studies including the elucidation of their activation profile, interaction proteins and phenotypic changes of transgenic plants will be helpful to clarify the accurate biological role of each member of *GmSnRK2* family in vivo.

The dynamic change of gene expression could provide useful information of gene functions. Through re-analysis of public RNA-Seq datasets, we found that *SnRK2* genes exhibited diverse spatio-temporal expression patterns during the growth and development of different tissues in soybean. In *Arabidopsis*, *SRK2B* of Group 1 was expressed in various tissues, and *SRK2F* of Group 2 was mainly expressed in flowers [45,46]. Soybean and *Arabidopsis SnRK2* genes in the same “Group” showed similar tissue and developmental stage-specific expression patterns. *GmSnRK2.2* and *GmSnRK2.16* of Group 4 were preferentially expressed in meristem. Notably, *GmSnRK2s* of Group 1, such as *GmSnRK2.7*, *GmSnRK2.14*, *GmSnRK2.15* and *GmSnRK2.18*, showed specific expression patterns during seed development. *GmSnRK2.14* and *GmSnRK2.15* were highly expressed in the late stages of seed development, while *GmSnRK2.7* and *GmSnRK2.18* were highly expressed in the early and mid-stages respectively, which indicated that members of Group 1 might play different roles during seed development in soybean.

Strigolactones (SLs), as the most recently characterized plant hormones, could stimulate the branching and play important roles in the establishment of a symbiotic association between the plant and fungi in the rhizosphere [47]. To our knowledge, there were no reports regarding the interaction between SLs and *SnRK2s*. Our study revealed that some *SnRK2* genes were strongly induced in soybean by SLs. Interestingly, *GmSnRK2.6* did not respond to NaCl and ABA treatments, but was differentially expressed after SLs treatment, which indicated that *GmSnRK2.6* might be specifically involved in SLs signaling pathway. Further elucidation of these SL-responsive *SnRK2s* might be helpful for scientists to reveal the molecular mechanisms underlying the interactions of between SLs and other different pathways.

In *Brassica rapa* ssp. *Chinensis*, some *SnRK2* genes, such as *BcSnRK2.1a*, *BcSnRK2.1b*, *BcSnRK2.4b* and *BcSnRK2.8b*, showed close correlations in expression patterns under ABA and low temperature treatment [22]. Similarly, 10, 18 and 14 *GmSnRK2s* showed significant correlations in the changes of expression in the ABA-related, NaCl-related and SLs-related co-regulatory networks, respectively. Among them, the largest count of *GmSnRK2* genes belongs to Group 3. Most of the members of Group 3, including *GmSnRK2.1*, *GmSnRK2.3*, *GmSnRK2.9*, *GmSnRK2.12*, *GmSnRK2.17* and *GmSnRK2.21*, contained ABREs as well as various *cis*-active response elements, such as AuxRR-core, P-box, TCA-element, CGTCA-motif, TGACG-motif, ERE, GARE-motif, TATC-box, Circadian, low-temperature responsiveness (LTR), MBS, TC-rich repeats, wound-responsive element (WUN-motif) and Box-W1. Furthermore, the co-regulated *GmSnRK2* genes with significant correlations shared similar *cis*-active elements in their promoters, which suggested that these elements might be responsible for stress-responsive co-expression of *GmSnRK2s* under different environmental conditions. Notably, there were common correlations of *GmSnRK2* genes under different treatments, such as the positive correlations among *GmSnRK2.3*, *GmSnRK2.8*, and *GmSnRK2.9* under ABA and NaCl treatments, the positive correlation between *GmSnRK2.11* and *GmSnRK2.17* under NaCl and SLs treatments, which indicated that these *GmSnRK2s* might exhibit synergistic effects of signal regulation in response to various stresses. Intriguingly, the paralogous gene pairs of *GmSnRK2s* showed no significant correlations with each other in the ABA-related, NaCl-related, and SLs-related co-regulated networks, which suggested that the paralogous *GmSnRK2* genes exhibited functional divergence during the evolution of soybean. Gene co-regulatory networks play important roles in the growth and developmental processes for organisms [48]. Of 22 *GmSnRK2* genes, 16 *SnRK2s* showed significant correlations in expression during seed development. *GmSnRK2.5* and *GmSnRK2.20* showed negative correlation in both seed development-related and ABA-related co-regulatory networks. By contrast, *GmSnRK2.3* and *GmSnRK2.8* had positive correlation under salt stress and ABA treatments, but had negative correlation during seed development. These results suggested that the correlations between *GmSnRK2s* varied under different conditions.

In this study, we presented a genome-wide identification and characterization of *GmSnRK2* in soybean, in which gene structures, phylogenetic relation, *cis*-acting regulatory elements and expression patterns of *GmSnRK2s* in different tissues or treatments, were analyzed in comparison with two model plants, *Arabidopsis* and rice. Our study provides useful information for scientists to further elucidate the function of *GmSnRK2* in soybean.

## 4. Materials and Methods

### 4.1. Plant Materials

Seeds of *Glycine max* L. *Merr* were germinated in vermiculite saturated with water. One week-old seedlings were transferred to a hydroponic system with half-strength Hoagland’s nutrient solution and grown in an incubator at 28 °C under a 16:8 (light:dark) photoperiod. After three weeks, for the gene expression analyses using qRT-PCR under salt, ABA and *rac*-GR24 stress, the seedlings were treated with half-strength Hoagland’s solution containing 150 mM NaCl, 100 μM ABA (Sigma-Aldrich, St. Louis, MO, USA) and 10 μM *rac*-GR24 (Sigma-Aldrich), respectively, at four time points (0, 8, 16 and 24 h). The seedlings with no treatment were used as a control. For all conditions, including control, three biological replicates were used. The treatments were performed at 0 h when the light period started. Leaves were collected at each time point, and immediately frozen in liquid nitrogen and stored at −80 °C.

### 4.2. Identification of GmSnRK2 Genes and Gene Structure Analysis

The genome sequence and gene annotation of soybean (*Glycine max* L. *Merr*) were downloaded from the Joint Genome Institute (JGI) database (available online: https://phytozome.jgi.doe.gov/pz/portal.html#!info?alias=Org_Gmax). The peptides of SnRK2 proteins of *Arabidopsis thaliana* and *Oryza sativa* were used as queries in a TBLASTN (version 2.2.26, Bethesda, MD, USA) search against the soybean genome with an *E*-value threshold of 1 × 10^−5^. The accession numbers or locus IDs of *SnRK2* genes of *Arabidopsis thaliana* and *Oryza sativa* were listed in supplementary materials (Table S1). DNA sequences of soybean genomic regions matching to *AtSnRK2s* and *OsSnRK2s* with high identity were extracted and used to infer the exon-intron structures of the *GmSnRK2* genes by GeneWise2 (version 2.4.1, Hinxton, Cambridge, UK ) [49]. The protein sequences of the predicted GmSnRK2s were queried against all the annotated proteins of *Arabidopsis thaliana* and *Oryza sativa* using BLASTP (version 2.2.26, Bethesda, MD, USA). The putative GmSnRK2s with best hits of the *Arabidopsis thaliana* and *Oryza sativa* SnRK2s were remained, and further queried against the annotated genes of soybean in JGI database. According to the gene structures of predicted *GmSnRK2s* and their best hits in database, the exon-intron structures of the *GmSnRK2s* were refined, and the start/stop codon, 5′-unstranslated region (UTR) and 3′-UTR were also revised.

### 4.3. Functional Domain and Cis-Acting Regulatory Elements Analysis

The peptides of GmSnRK2 proteins were translated from coding DNA sequences with standard genetic code, and were further inspected and revised according to the alignment with soybean protein sequences in JGI database by BLASTP. The peptides of GmSnRK2s were submitted to InterProScan (version 5.25-64.0, Hinxton, Cambridge, UK) to examine the presence and completeness of the protein kinase domain. The physical and chemical properties including molecular weight, isoelectric point, aliphatic index, and GRAVY were calculated using Protparam (available online: http://web.expasy.org/protparam/). The upstream 2 kb regions from transcription start site (TSS) of each *GmSnRK2* genes were extracted from soybean genome sequence, and used to identify *cis*-acting regulatory elements by PlantCARE server (available online: http://bioinformatics.psb.ugent.be/webtools/plantcare/html/) [50].

### 4.4. Multiple Sequence Alignment and Phylogenetic Analysis

The protein sequences of GmSnRK2s, AtSnRK2s and OsSnRK2s were used to perform multiple sequence alignment using MUSCLE (version 3.2, Hinxton, Cambridge, UK) [51]. Phylogenetic tree was inferred using the Neighbor-Joining (NJ) method available in MEGA5 (version 5.1 β, Hachioji, Tokyo, Japan) [52]. The robustness of each node in the tree was determined using 1000 bootstrap replicates.

### 4.5. Expression Analysis

The publicly available transcriptome dataset of soybean (accession number: SRP038111; March 2014) was downloaded from the National Center for Biotechnology Information (NCBI) Sequence Read Archive (SRA) database, and used for gene expression profiling [53]. Mapping of these RNA-Seq reads was done by Tophat2 (version 2.1.0, Baltimore, MD, USA) with the default settings [54]. Quantification of gene expression was done using the Fragments Per Kilobase Of Exon Per Million Fragments Mapped (FPKM) algorithm. The clustered heatmap of Z scaled resulting FPKM values per tissue for *GmSnRK2s* was generated using the pheatmap function of R package (version 3.2.2; available online: https://cran.r-project.org/web/packages/pheatmap/).

### 4.6. RNA Extraction and cDNA Synthesis

Total RNAs were extracted from each leaf sample using TRIzol reagent (Invitrogen, Carlsbad, CA, USA) following the manufacturer’s procedure. The quantity/quality of total RNA was measured with a NanoDrop^®^ 1000 Spectrophotometer (Wilmington, DE, USA). Two μg of total RNA was used to synthesize copy DNA (cDNA) using TaKaRa PrimerScriptTM RT reagent kit with gDNA Eraser (Takara, Shiga, Japan) according to the manufacturer’s protocol.

### 4.7. Quantitative Real-Time Polymerase Chain Reaction

The expression analysis of the 22 *GmSnRK2* genes was performed by qRT-PCR using a SYBR Green PCR kit (GeneCopoeia, Inc., Rockville, MD, USA) with ViiA^TM^ 7 Dx platform (ABI, Los Angeles, CA, USA). The amplified primers and internal controls were listed in supplementary materials (Table S2). The qRT-PCR reaction condition was as following: 95 °C for 30 s, 40 cycles at 95 °C for 5 s, 58 °C for 30 s, and 72 °C for 30 s. After amplification, the melting curve and amplification curve were checked to evaluate specific amplification. The relative expression levels of these genes were analyzed by the 2^−ΔΔ*C*t^ method, and the *GmSKIP* gene was used as the internal control. All qRT-PCR reactions were assayed in triplicates.

### 4.8. Pearson Correlation Analysis

Pearson correlation coefficients (PCCs) and *p*-value of expression levels of *GmSnRK2* gene pairs were calculated using R package program based on the RNA-Seq and qRT-PCR results. Gene co-regulatory networks were constructed by Cytoscape (version 3.1, Seattle, WA, USA) based on the PCCs of *GmSnRK2* gene pairs with a *p*-value significance level of 0.05 [55].

## Figures and Tables

**Table 1:** Soybean *SnRK2* gene family.

Gene Name	Locus Name	Gene Location ^1^	Transcript Length (bp)	Protein Length (aa)	Molecular Weight (kDa)	Isoelectric Point (pI)	Grand Average of Hydropathy (GRAVY)	Best Hits of *Arabidopsis* and *Rice*	Domain (Start–End aa) ^2^
*GmSnRK2.1*	Glyma01g39020	Chr01:50978690–50990291: −	2056	359	40.83	4.69	−0.245	*AtSRK2I*; *OsSAPK10*	IPR017441 (27–50); IPR008271 (136–148)
*GmSnRK2.2*	Glyma01g41260 (*SPK1*)	Chr01:52822191–52826139: +	1432	339	38.56	6.44	−0.432	*AtSRK2E*; *OsSAPK3*	IPR017441 (11–34); IPR008271 (120–132)
*GmSnRK2.3*	Glyma02g15330	Chr02:13828174–13832341: +	1813	354	40.21	5.04	−0.359	*AtSRK2E*; *OsSAPK10*	IPR017441 (24–47); IPR008271 (133–145)
*GmSnRK2.4*	Glyma02g37090	Chr02:42422548–42426147: −	1577	338	38.3	5.38	−0.267	*AtSRK2C*; *OsSAPK2*	IPR017441 (10–33); IPR008271 (119–131)
*GmSnRK2.5*	Glyma04g38270 (*SPK4*)	Chr04:44680353–44684054: +	1763	349	40.35	6.1	−0.497	*AtSRK2A*; *OsSAPK7*	IPR017441 (10–33); IPR008271 (119–131)
*GmSnRK2.6*	Glyma05g31000	Chr05:36237913–36240990: +	1376	309	35.37	5.17	−0.366	*AtSRK2C*; *OsSAPK2*	IPR017441 (10–33); IPR008271 (85–97)
*GmSnRK2.7*	Glyma05g33170	Chr05:37897658–37901759: −	2020	351	40.43	5.95	−0.523	*AtSRK2A*; *OsSAPK7*	IPR017441 (10–33); IPR008271 (119–131)
*GmSnRK2.8*	Glyma05g05540	Chr05:4967484–4972474: −	1532	336	38.12	5.6	−0.447	*AtSRK2E*; *OsSAPK3*	IPR017441 (11–34); IPR008271 (120–132)
*GmSnRK2.9*	Glyma05g09460	Chr05:9361881–9367360: −	1922	360	40.8	4.75	−0.268	*AtSRK2I*; *OsSAPK10*	IPR017441 (29–52); IPR008271 (138–150)
*GmSnRK2.10*	Glyma06g16780	Chr06:13183468–13187110: −	1648	346	39.98	6.48	−0.468	*AtSRK2A*; *OsSAPK7*	IPR017441 (10–33); IPR008271 (119–131)
*GmSnRK2.11*	Glyma07g29500	Chr07:34352503–34356772: −	2034	364	41.46	4.93	−0.334	*AtSRK2E*; *OsSAPK10*	IPR017441 (29–52); IPR008271 (138–150)
*GmSnRK2.12*	Glyma07g33120	Chr07:38032643–38037084: −	1769	353	40.07	5.04	−0.344	*AtSRK2E*; *OsSAPK10*	IPR017441 (24–47); IPR008271 (133–145)
*GmSnRK2.13*	Glyma08g14210	Chr08:10340382–10343745: +	1701	345	39.24	5.15	−0.3	*AtSRK2E*; *OsSAPK1*	IPR017441 (10–33); IPR008271 (119–131)
*GmSnRK2.14*	Glyma08g20090	Chr08:15173104–15177102: −	1959	352	40.26	6.1	−0.486	*AtSRK2B*; *OsSAPK7*	IPR017441 (10–33); IPR008271 (119–131)
*GmSnRK2.15*	Glyma08g00770 (*SPK3*)	Chr08:400210–404391: −	2076	351	40.39	5.75	−0.529	*AtSRK2A*; *OsSAPK7*	IPR017441 (10–33); IPR008271 (119–131)
*GmSnRK2.16*	Glyma11g04150 (*SPK2*)	Chr11:2759687–2763609: −	1585	339	38.66	6.39	−0.45	*AtSRK2E*; *OsSAPK3*	IPR017441 (11–34); IPR008271 (120–132)
*GmSnRK2.17*	Glyma11g06250	Chr11:4436484–4443383: +	2211	359	40.78	4.68	−0.234	*AtSRK2I*; *OsSAPK10*	IPR017441 (27–50); IPR008271 (136–148)
*GmSnRK2.18*	Glyma12g29130	Chr12:32505082–32508220: +	1719	359	41.08	5.82	−0.537	*AtSRK2B*; *OsSAPK7*	IPR017441 (10–33); IPR008271 (119–131)
*GmSnRK2.19*	Glyma14g35380	Chr14:44313521–44317470: −	1831	338	38.18	5.38	−0.251	*AtSRK2C*; *OsSAPK1*	IPR017441 (10–33); IPR008271 (119–131)
*GmSnRK2.20*	Glyma17g15860	Chr17:12585658–12589679: −	1405	336	38.04	5.71	−0.46	*AtSRK2E*; *OsSAPK3*	IPR017441 (11–34); IPR008271 (120–132)
*GmSnRK2.21*	Glyma17g20610	Chr17:19751156–19756370: +	1796	360	40.91	4.75	−0.273	*AtSRK2I*; *OsSAPK10*	IPR017441 (29–52); IPR008271 (138–150)
*GmSnRK2.22*	Glyma20g01240	Chr20:846957–851473: −	2064	364	41.49	4.89	−0.344	*AtSRK2E*; *OsSAPK10*	IPR017441 (29–52); IPR008271 (138–150)

^1^ Chromosome:start position–end position: strand, (–) means antisense strand of chromosome; (+) means positive-sense strand of chromosome. ^2^ Protein kinase, adenosine triphosphate (ATP) binding site (IPR017441); Serine/threonine-protein kinase, active site (IPR008271). bp: base pair; aa: amino acid.

**Table 2:** Phytohormone response elements in the promoters of *GmSnRK2s*.

Group	Gene Name	*Cis* Elements	Number	Sequence Pattern (5′-3′)	Position (Strand)	Function
Group 1	*GmSnRK2.5*	CGTCA-motif	1	CGTCA	−410 bp (−)	MeJA Response Element
		ERE	3	ATTTCAAA	−526 bp (−); −911 bp (+); −750 bp (+)	Ethylene Response element
		P-box	2	CCTTTTG; GCCTTTTGAGT	−68 bp (−); −308 bp (−)	Gibberellin Response Element
		TGACG-motif	1	TGACG	−410 bp (+)	MeJA Response Element
	*GmSnRK2.7*	CGTCA-motif	2	CGTCA	−717 bp (−); −834 bp (−)	MeJA Response Element
		TGACG-motif	2	TGACG	−717 bp (+); −834 bp (+)	MeJA Response Element
		GARE-motif	1	TCTGTTG	−293 bp (−)	Gibberellin Response Element
		TCA-element	1	CCATCTTTTT	−1412 bp (−)	Salicylic acid Response Element
		TGA-element	2	AACGAC	−836 bp (−); −1438 bp (+)	Auxin Response Element
	*GmSnRK2.10*	ERE	1	ATTTCAAA	−359 bp (−)	Ethylene Response element
	*GmSnRK2.14*	ERE	1	ATTTCAAA	−211 bp (+)	Ethylene Response element
		TATC-box	1	TATCCCA	−1289 bp (−)	Gibberellin Response Element
	*GmSnRK2.15*	AuxRR-core	1	GGTCCAT	−1409 bp (−)	Auxin Response Element
		ERE	1	ATTTCAAA	−972 bp (−)	Ethylene Response element
		TGA-element	1	AACGAC	−559 bp (+)	Auxin Response Element
	*GmSnRK2.18*	ABRE	1	TACGTG	−516 bp (+)	ABA Response Element
		CGTCA-motif	1	CGTCA	−1228 bp (−)	MeJA Response Element
		TGACG-motif	1	TGACG	−1228 bp (+)	MeJA Response Element
		ERE	2	ATTTCAAA	−394 bp (+); −628 bp (+)	Ethylene Response element
		TCA-element	2	GAGAAGAATA; CCATCTTTTT	−325 bp (−); −377 bp (+)	Salicylic acid Response Element
		TGA-element	1	AACGAC	−837 bp (+)	Auxin Response Element
Group 2	*GmSnRK2.4*	ABRE	1	CACGTG	−1258 bp (−)	ABA Response Element
		GARE-motif	1	AAACAGA	−1347 bp (−)	Gibberellin Response Element
	*GmSnRK2.6*	GARE-motif	1	AAACAGA	−314 bp (+)	Gibberellin Response Element
		TCA-element	3	CAGAAAAGGA; CCATCTTTTT; CAGAAAAGGA	−317 bp (+); −1352 bp (−); −564 bp (+)	Salicylic acid Response Element
	*GmSnRK2.13*	ABRE	2	GACACGTGGC; TACGTG	−657 bp (−); −1212 bp (−)	ABA Response Element
		ERE	2	ATTTCAAA	−99 bp (−); −1336 bp (+)	Ethylene Response element
		P-box	1	GCCTTTTGAGT	−510 bp (+)	Gibberellin Response Element
		TGA-element	1	AACGAC	−84 bp (+)	Auxin Response Element
	*GmSnRK2.19*	ABRE	1	CACGTG	−1401 bp (−)	ABA Response Element
		GARE-motif	1	AAACAGA	−1483 bp (−)	Gibberellin Response Element
Group 3	*GmSnRK2.1*	ABRE	1	TACGTG	−79 bp (+)	ABA Response Element
		AuxRR-core	1	GGTCCAT	−1158 bp (−)	Auxin Response Element
		P-box	1	CCTTTTG	−115 bp (−)	Gibberellin Response Element
		TCA-element	2	CCATCTTTTT; GAGAAGAATA	−164 bp (−); −412 bp (−)	Salicylic acid Response Element
	*GmSnRK2.3*	CGTCA-motif	3	CGTCA	−261 bp (+); −1314 bp (+); −295 bp (+)	MeJA Response Element
		TGACG-motif	3	TGACG	−261 bp (−); −1314 bp (−); −295 bp (−)	MeJA Response Element
		ERE	1	ATTTCAAA	−481 bp (−)	Ethylene Response element
		GARE-motif	1	AAACAGA	−62 bp (+)	Gibberellin Response Element
		P-box	1	CCTTTTG	−1451 bp (−)	Gibberellin Response Element
		TCA-element	1	GAGAAGAATA	−934 bp (+)	Salicylic acid Response Element
	*GmSnRK2.9*	ABRE	1	CCGCGTAGGC	−1360 bp (−)	ABA Response Element
		CGTCA-motif	1	CGTCA	−1386 bp (+)	MeJA Response Element
		ERE	1	ATTTCAAA	−334 bp (−)	Ethylene Response element
		GARE-motif	1	AAACAGA	−403 bp (−)	Gibberellin Response Element
	*GmSnRK2.11*	TCA-element	2	CCATCTTTTT	−1352 bp (+); −1469 bp (−)	Salicylic acid Response Element
	*GmSnRK2.12*	ABRE	2	CACGTG; TACGTG	−538 bp (+); −1147 bp (−)	ABA Response Element
		AuxRR-core	1	GGTCCAT	−1345 bp (+)	Auxin Response Element
		CGTCA-motif	2	CGTCA	−117 bp (+); −1361 bp (+)	MeJA Response Element
		TGACG-motif	2	TGACG	−117 bp (−); −1361 bp (−)	MeJA Response Element
		TCA-element	2	CCATCTTTTT	−98 bp (−); −710 bp (+)	Salicylic acid Response Element
	*GmSnRK2.17*	ABRE	2	CACGTG; AGTACGTGGC	−207 bp (+); −410 bp (−)	ABA Response Element
		AuxRR-core	1	GGTCCAT	−981 bp (−)	Auxin Response Element
		ERE	1	ATTTCAAA	−371 bp (−)	Ethylene Response element
		GARE-motif	1	AAACAGA	−126 bp (−)	Gibberellin Response Element
		TATC-box	1	TATCCCA	−1305 bp (+)	Gibberellin Response Element
		TCA-element	1	CCATCTTTTT	−780 bp (+)	Salicylic acid Response Element
	*GmSnRK2.21*	ABRE	3	CCTACGTGGC; ACGTGGC; TACGTG	−1349 bp (+); −1352 bp (+); −1351 bp (+)	ABA Response Element
		CGTCA-motif	1	CGTCA	−1374 bp (+)	MeJA Response Element
		ERE	1	ATTTCAAA	−309 bp (−)	Ethylene Response element
		TGACG-motif	1	TGACG	−1374 bp (−)	MeJA Response Element
	*GmSnRK2.22*	TGACG-motif	1	TGACG	−705 bp (−)	MeJA Response Element
		CGTCA-motif	1	CGTCA	−705 bp (+)	MeJA Response Element
		TCA-element	2	GAGAAGAATA; TCAGAAGAGG	−425 bp (+); −792 bp (+)	Salicylic acid Response Element
Group 4	*GmSnRK2.2*	CGTCA-motif	1	CGTCA	−477 bp (−)	MeJA Response Element
		TGACG-motif	1	TGACG	−477 bp (+)	MeJA Response Element
		TCA-element	1	CCATCTTTTT	−82 bp (+)	Salicylic acid Response Element
	*GmSnRK2.16*	CGTCA-motif	1	CGTCA	−865 bp (−)	MeJA Response Element
		TGACG-motif	1	TGACG	−865 bp (+)	MeJA Response Element
		TCA-element	1	CCATCTTTTT	−449 bp (+)	Salicylic acid Response Element
	*GmSnRK2.20*	GARE-motif	1	AAACAGA	−281 bp (+)	Gibberellin Response Element

MeJA: Methyl Jasmonic acid; GARE: Gibberellin response element.

## Supplementary Materials

Supplementary materials can be found at www.mdpi.com/1422-0067/18/9/1834/s1.

## Abbreviations

ABA	Abscisic acid
SLs	Strigolactones
MeJA	Methyl Jasmonic acid
ERE	Ethylene response element
JGI	Joint Genome Institute
LTR	Low-temperature responsiveness
MBS	MYB binding site involved in drought-inducibility
GARE	Gibberellin response element
HSE	Heat stress responsiveness
WUN-motif	Wound-responsive element
qRT-PCR	Quantitative real-time polymerase chain reaction
FPKM	Fragments Per Kilobase Of Exon Per Million Fragments Mapped
GRAVY	Grand average of hydropathy
PCCs	Pearson correlation coefficients
CDS	Coding sequence
TSS	Transcription start site
MW	Molecular weight
pI	Isoelectric point
aa	Amino acid
bp	Base pair
kDa	Kilodalton
