# Supplementary material for: Genome-Wide Identification and Characterization of the GmSnRK2 Family in Soybean
Source: Int J Mol Sci. 2017 Aug 23;18(9):1834. doi: 10.3390/ijms18091834 (PMC5618483; doi:10.3390/ijms18091834)
Supplement: Supplementary file 1 [file ijms-18-01834-s001.pdf]

Genome–Wide Identification and Characterization of the *GmSnRK2* Family in Soybean

Wei Zhao [†], Yi–Hui Cheng [†], Chi Zhang, Xin–Jie Shen, Qing–Bo You, Wei Guo, Xiang Li, Xue–Jiao Song, Xin–An Zhou and Yong–Qing Jiao [*]

Table S1. List of AtSnRK2s and OsSnRK2s.

Taxa Name	Gene name	Accession number or locus ID	Uniprot	Proten length (aa)
Arabidopsis thaliana	*AtSRK2H*	AT5G63650	Q9FFP9	360
	AtSRK2J	AT2G23030	O64812	339
	AtSRK2A	AT1G10940	P43291	363
	AtSRK2B	AT1G60940	Q9C958	361
	AtSRK2F	AT4G40010	Q9SMQ4	350
	AtSRK2C	AT1G78290	Q9M9E9	343
	AtSRK2I	AT5G66880	Q39193	361
	AtSRK2D	AT3G50500	Q39192	362
	AtSRK2E	AT4G33950	Q940H6	362
	AtSRK2G	AT5G08590	P43292	353
Oryza sativa	*OsSAPK6*	LOC_Os02g34600	Q6ZI44	365
	OsSAPK4	LOC_Os01g64970	F6M7C8	360
	OsSAPK5	LOC_Os04g59450	Q7XKA8	370
	OsSAPK2	LOC_Os07g42940	Q0D4J7	339
	OsSAPK3	LOC_Os10g41490	P0C5D6	334
	OsSAPK1	LOC_Os03g27280	Q75LR7	342
	OsSAPK8	LOC_Os03g55600	F6M7D2	371
	OsSAPK10	LOC_Os03g41460	Q75H77	362
	OsSAPK9	LOC_Os12g39630	Q75V57	361
	OsSAPK7	LOC_Os04g35240	Q7XQP4	359

Table S2. List of primers for qRT–PCR.

Gene name	Forward primer（5' > 3'）	Reverse primer（5' > 3'）
GmSnRK2.1	GGTCCCCGCAGCTGGTATG	TGACCCTGCAGAGAGAATCACTATT
GmSnRK2.2	CGTAGTCGGGTATGGCGTAT	ATGTTTGGTCGTGTGGTGTG
GmSnRK2.3	CAGCAACAAGTTCCTCAGTATGTT	TGTTGGGCCAGGAATGGATTT
GmSnRK2.4	TCAGCATCAAAGTCATCCAGG	GCCCATGGAACTGACGGA
GmSnRK2.5	GGGTCATCCTGGTCCTCAAA	TCATCTTTGCTGCACTCACG
GmSnRK2.6	GCACAGACAAAATCACCGCTT	TGCAAAATGATGATGTGAATGACG
GmSnRK2.7	GCCTAGATACTGGAGGAGGG	TGTAGCAAATCCATTAAGGAGAATA
GmSnRK2.8	GTCCAACTTCTCCTGCTTTGG	CCAAGAGGATCACCATCCCG
GmSnRK2.9	CTTTAAACGAGGTGCCGAGC	ACTGAGGATGAGGCTCGATT
GmSnRK2.10	ATCATCCTGGTCCTCAAAGGG	CTGCATTCGCGACCGAAATC
GmSnRK2.11	TCGTCATCAATGTCCAAGCTA	GAACACGATGAACAGGCAA
GmSnRK2.12	TCTATCTGCTTTTCACCTCTCTCA	CCGGGAATGGATTTGCCGAT
GmSnRK2.13	GCAAGATGAGTTGGAGTAAGC	CAGGAAACTTTGGTGTGGCA
GmSnRK2.14	TCATCTTCTTCAGTCTCAGCCA	GCTCAAGCTGCCTACTACAG
GmSnRK2.15	CCTCATCACCACGCACAAC	GGCTGAAAGAAGGGGTTACG
GmSnRK2.16	CCTCATCACCACGCACAAC	GGCTGAAAGAAGGGGTTACG
GmSnRK2.17	GTCCCTGCAGCGGGTAT	TGCAGAGAGAATCACTATTCCTGA
GmSnRK2.18	CTTCTTCAGCCTCGGTCACT	TTGTTGAGGAGGCCAAGACT
GmSnRK2.19	CATGCTGCCCCCAATAAGAAGG	AGGAACTTGCCCATAGAACAGATG
GmSnRK2.20	GAATTGAAGATACATACCTTGGCA	TGGTGCATACCCATTTGAAGAT
GmSnRK2.21	ATCCTCGGTAAAACGCCCA	ATAGAGGAGGGGCAATCACATT
GmSnRK2.22	CCGTCTCTAGGTCCTCATCC	GCCTGATCAACCAATGCAGA
GmSKIP	GAGCCCAAGACATTGCGAGAG	CGGAAGCGAAGAACTGAACC

Table S3. Defense and stress response elements in the promoters of *GmSnRK2s*.

Gene Name	*Cis* Elements	Number	Sequence Pattern	Position (Strand)	Function
GmSnRK2.1	TC-rich repeats	3	GTTTTCTTAC; ATTTTCTTCA; ATTCTCTAAC	−610 bp (−); −916 bp (+); −790 bp (+)	*Cis-acting* element involved in defense and stress responsiveness
GmSnRK2.2	Circadian	1	CAANNNNATC	−812 bp (−)	*Cis-acting* regulatory element involved in circadian control
	TC-rich repeats	2	GTTTTCTTAC; ATTTTCTTCA	−226 bp (+); −1364 bp (+)	*Cis-acting* element involved in defense and stress responsiveness
	HSE	3	AAAAAATTTC	−126 bp (+); −919 bp (−); −309 bp (−)	*Cis-acting* element involved in heat stress responsiveness
	WUN-motif	1	AAATTTCCT	−917 bp (−)	wound−responsive element
GmSnRK2.3	Circadian	3	CAANNNNATC	−227 bp (−); −1451 bp (+); −980 bp (+)	*Cis-acting* regulatory element involved in circadian control
	LTR	1	CCGAAA	−1332 bp (−)	*Cis-acting* element involved in low−temperature responsiveness
	MBS	1	CAACTG	−1146 bp (+)	MYB binding site involved in drought−inducibility
	TC-rich repeats	3	ATTTTCTTCA; ATTTTCTCCA	−322 bp (+); −451 bp (+); −690 bp (+)	*Cis-acting* element involved in defense and stress responsiveness
	WUN-motif	1	TCATTACGAA	−988 bp (+)	wound−responsive element
GmSnRK2.4	Circadian	1	CAANNNNATC	−1144 bp (+)	*Cis-acting* regulatory element involved in circadian control
	ELI−box3	2	AAACCAATT	−21 bp (−); −144 bp (+)	elicitor−responsive element
	TC-rich repeats	2	ATTTTCTCCA; ATTTTCTTCA	−188 bp (+); −410 bp (+)	*Cis-acting* element involved in defense and stress responsiveness
GmSnRK2.5	Circadian	1	CAANNNNATC	−1058 bp (+)	*Cis-acting* regulatory element involved in circadian control
	TC-rich repeats	2	ATTTTCTTCA	−385 bp (+); −596 bp (+)	*Cis-acting* element involved in defense and stress responsiveness
GmSnRK2.6	HSE	1	AAAAAATTTC	−2 bp (−)	*Cis-acting* element involved in heat stress responsiveness
	MBS	1	CAACTG	−1452 bp (−)	MYB binding site involved in drought−inducibility
	TC-rich repeats	2	ATTTTCTTCA;	−897 bp (−); −1047 bp (+)	*Cis-acting* element involved in defense and stress

			ATTCTCTAAC		responsiveness
GmSnRK2.7	Box-W1	1	TTGACC	−345 bp (+)	fungal elicitor responsive element
	HSE	1	AAAAAATTTC	−1222 bp (−)	*Cis-acting* element involved in heat stress responsiveness
	LTR	1	CCGAAA	−180 bp (+)	*Cis-acting* element involved in low−temperature responsiveness
	TC-rich repeats	1	ATTTTCTTCA	−442 bp (−)	*Cis-acting* element involved in defense and stress responsiveness
GmSnRK2.8	HSE	3	AAAAAATTTC	−266 bp (+); −432 bp (−); −267 bp (+)	*Cis-acting* element involved in heat stress responsiveness
	TC-rich repeats	1	ATTTTCTTCA	−167 bp (−)	*Cis-acting* element involved in defense and stress responsiveness
GmSnRK2.9	Circadian	3	CAANNNNATC	−111 bp (+); −1482 bp (+); −175 bp (+)	*Cis-acting* regulatory element involved in circadian control
	Box-W1	1	TTGACC	−898 bp (+)	fungal elicitor responsive element
	MBS	2	CAACTG	−1463 bp (−); −1046 bp (+)	MYB binding site involved in drought−inducibility
	TC-rich repeats	1	ATTCTCTAAC	−943 bp (−)	*Cis-acting* element involved in defense and stress responsiveness
GmSnRK2.10	Circadian	3	CAANNNNATC	−745 bp (−); −1104 bp (+); −1024 bp (+)	*Cis-acting* regulatory element involved in circadian control
	HSE	1	AAAAAATTTC	−924 bp (−)	*Cis-acting* element involved in heat stress responsiveness
	LTR	1	CCGAAA	−1483 bp (+)	*Cis-acting* element involved in low−temperature responsiveness
	MBS	1	TAACTG	−513 bp (−)	MYB binding site involved in drought−inducibility
	TC-rich repeats	2	GTTTTCTTAC; ATTTTCTCCA	−414 bp (+); −1051 bp (+)	*Cis-acting* element involved in defense and stress responsiveness
GmSnRK2.11	HSE	1	AAAAAATTTC	−1247 bp (+)	*Cis-acting* element involved in heat stress responsiveness
	MBS	1	CAACTG	−1184 bp (+)	MYB binding site involved in drought−inducibility
	TC-rich repeats	1	ATTTTCTCCA	−1052 bp (−)	*Cis-acting* element involved in defense and stress responsiveness
GmSnRK2.12	Circadian	1	CAANNNNATC	−307 bp (+)	*Cis-acting* regulatory element involved in

					circadian control
	Box-W1	1	TTGACC	−734 bp (+)	fungal elicitor responsive element
	HSE	2	AAAAAATTTC	−585 bp (+); −630 bp (+)	*Cis-acting* element involved in heat stress responsiveness
	LTR	1	CCGAAA	−1378 bp (−)	*Cis-acting* element involved in low−temperature responsiveness
	MBS	1	CAACTG	−1205 bp (+)	MYB binding site involved in drought−inducibility
	TC-rich repeats	3	ATTTTCTCCA; GTTTTCTTAC	−366 bp (+); −690 bp (−); −815 bp (+)	*Cis-acting* element involved in defense and stress responsiveness
GmSnRK2.13	Circadian	2	CAANNNNATC	−58 bp (+); −586 bp (+)	*Cis-acting* regulatory element involved in circadian control
	Box-W1	1	TTGACC	−491 bp (−)	fungal elicitor responsive element
	HSE	2	AAAAAATTTC; AGAAAATTCG	−157 bp (−); −1022 bp (−)	*Cis-acting* element involved in heat stress responsiveness
	MBS	1	CAACTG	−500 bp (−)	MYB binding site involved in drought−inducibility
	TC-rich repeats	2	ATTTTCTTCA; ATTCTCTAAC	−1177 bp (−); −1323 bp (+)	*Cis-acting* element involved in defense and stress responsiveness
GmSnRK2.14	Circadian	1	CAANNNNATC	−1406 bp (−)	*Cis-acting* regulatory element involved in circadian control
	HSE	3	AAAAAATTTC	−99 bp (−); −258 bp (+); −153 bp (−)	*Cis-acting* element involved in heat stress responsiveness
	TC-rich repeats	3	ATTTTCTCCA; ATTTTCTTCA; ATTCTCTAAC	−102 bp (+); −805 bp (+); −538 bp (+)	*Cis-acting* element involved in defense and stress responsiveness
GmSnRK2.15	Circadian	2	CAANNNNATC	−927 bp (+); −1112 bp (+)	*Cis-acting* regulatory element involved in circadian control
	Box-W1	2	TTGACC	−616 bp (−); −656 bp (+)	fungal elicitor responsive element
	HSE	6	AAAAAATTTC; AGAAAATTCG	−15 bp (−); −1176 bp (−); −1236 bp (+); −73 bp (+); −404 bp (−); −403 bp (−)	*Cis-acting* element involved in heat stress responsiveness
	MBS	2	TAACTG	−498 bp (+); −532 bp (−)	MYB binding site involved in drought−inducibility
GmSnRK2.16	HSE	5	AAAAAATTTC	−20 bp (+); −459 bp (+); −68 bp (+); −55 bp (+); −81 bp (−)	*Cis-acting* element involved in heat stress responsiveness

	TC-rich repeats	1	ATTTTCTTCA	−596 bp (+)	*Cis-acting* element involved in defense and stress responsiveness
GmSnRK2.17	Circadian	1	CAANNNNATC	−1073 bp (+)	*Cis-acting* regulatory element involved in circadian control
	HSE	3	AAAAAATTTC	−663 bp (+); −1262 bp (+); −744 bp (+)	*Cis-acting* element involved in heat stress responsiveness
	MBS	1	TAACTG	−185 bp (−)	MYB binding site involved in drought−inducibility
	TC-rich repeats	1	ATTTTCTTCA	−1437 bp (−)	*Cis-acting* element involved in defense and stress responsiveness
GmSnRK2.18	Circadian	2	CAANNNNATC	−96 bp (+); −1340 bp (−)	*Cis-acting* regulatory element involved in circadian control
	Box-W1	1	TTGACC	−832 bp (−)	fungal elicitor responsive element
	HSE	2	AAAAAATTTC	−203 bp (+); −1271 bp (−)	*Cis-acting* element involved in heat stress responsiveness
	LTR	1	CCGAAA	−672 bp (−)	*Cis-acting* element involved in low−temperature responsiveness
	MBS	1	TAACTG	−451 bp (+)	MYB binding site involved in drought−inducibility
	TC-rich repeats	2	GTTTTCTTAC; ATTTTCTTCA	−456 bp (+); −1065 bp (−)	*Cis-acting* element involved in defense and stress responsiveness
GmSnRK2.19	TC-rich repeats	1	ATTTTCTCCA	−273 bp (−)	*Cis-acting* element involved in defense and stress responsiveness
GmSnRK2.20	Circadian	1	CAANNNNATC	−316 bp (+)	*Cis-acting* regulatory element involved in circadian control
	Box-W1	2	TTGACC	−23 bp (+); −626 bp (+)	fungal elicitor responsive element
	HSE	1	AAAAAATTTC	−768 bp (+)	*Cis-acting* element involved in heat stress responsiveness
	MBS	1	CAACTG	−1314 bp (+)	MYB binding site involved in drought−inducibility
	TC-rich repeats	1	GTTTTCTTAC	−821 bp (+)	*Cis-acting* element involved in defense and stress responsiveness
GmSnRK2.21	Circadian	4	CAANNNNATC	−85 bp (+); −771 bp (−); −149 bp (+); −1478 bp (+)	*Cis-acting* regulatory element involved in circadian control
	MBS	2	TAACTG; CAACTG	−550 bp (−); −1459 bp (−)	MYB binding site involved in drought−inducibility

	TC-rich repeats	1	ATTTTCTTCA	−1248 bp (−)	*Cis-acting* element involved in defense and stress responsiveness
GmSnRK2.22	HSE	1	AGAAAATTCG	−896 bp (+)	*Cis-acting* element involved in heat stress responsiveness
	MBS	1	CAACTG	−142 bp (−)	MYB binding site involved in drought−inducibility
	TC-rich repeats	2	ATTTTCTCCA	−1221 bp (+); −1481 bp (−)	*Cis-acting* element involved in defense and stress responsiveness

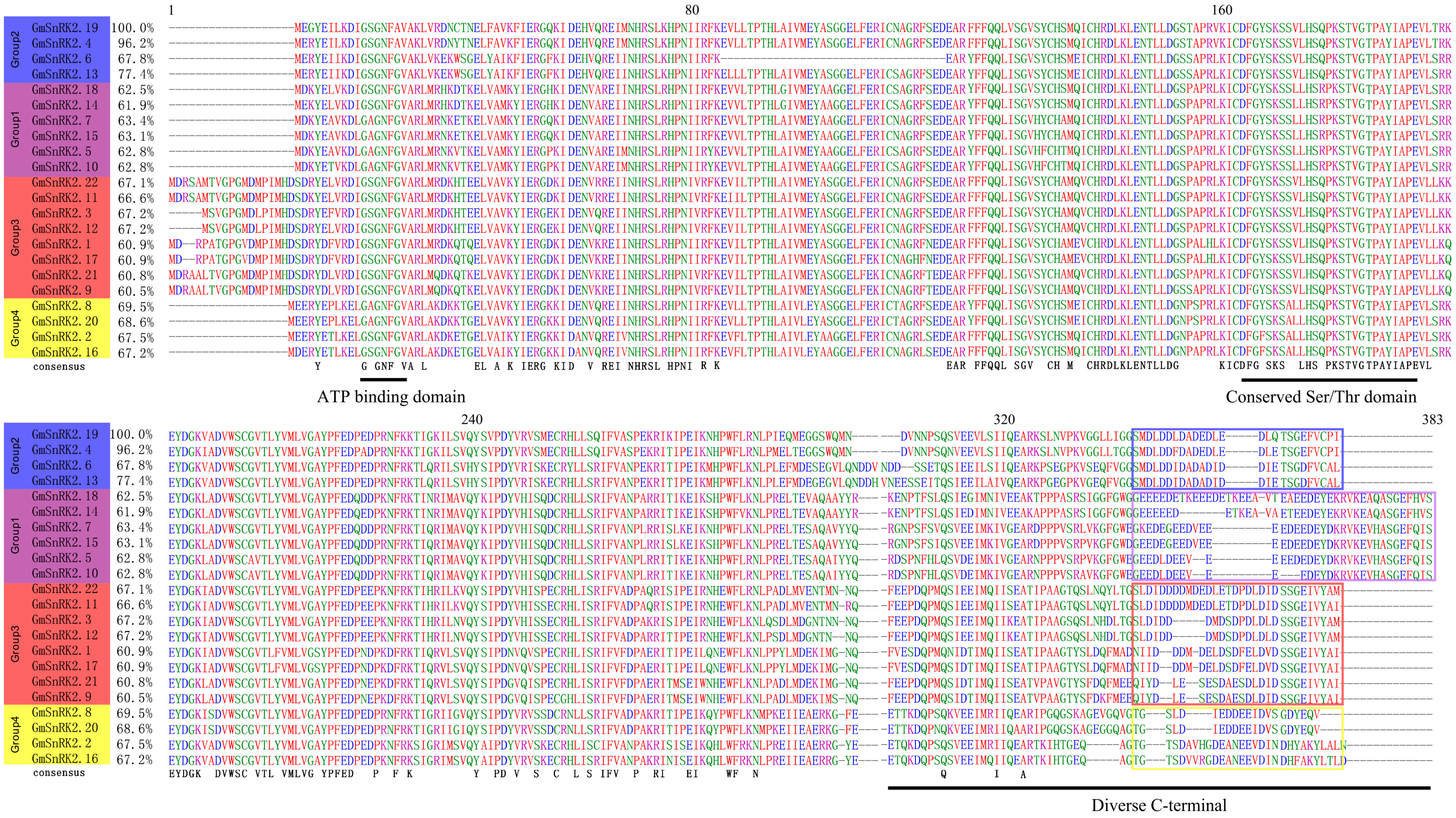

Figure S1. Multiple sequence alignment of GmSnRK2 proteins. ATP binding domain, converved Ser/Thr kinase domain, and diverse C–terminal were indicated by black lines at the bottom. The red, purple, blue and yellow boxes represented the C-terminal of GmSnRK2s from Group1, Group2, Group3 and Group4, respectively.

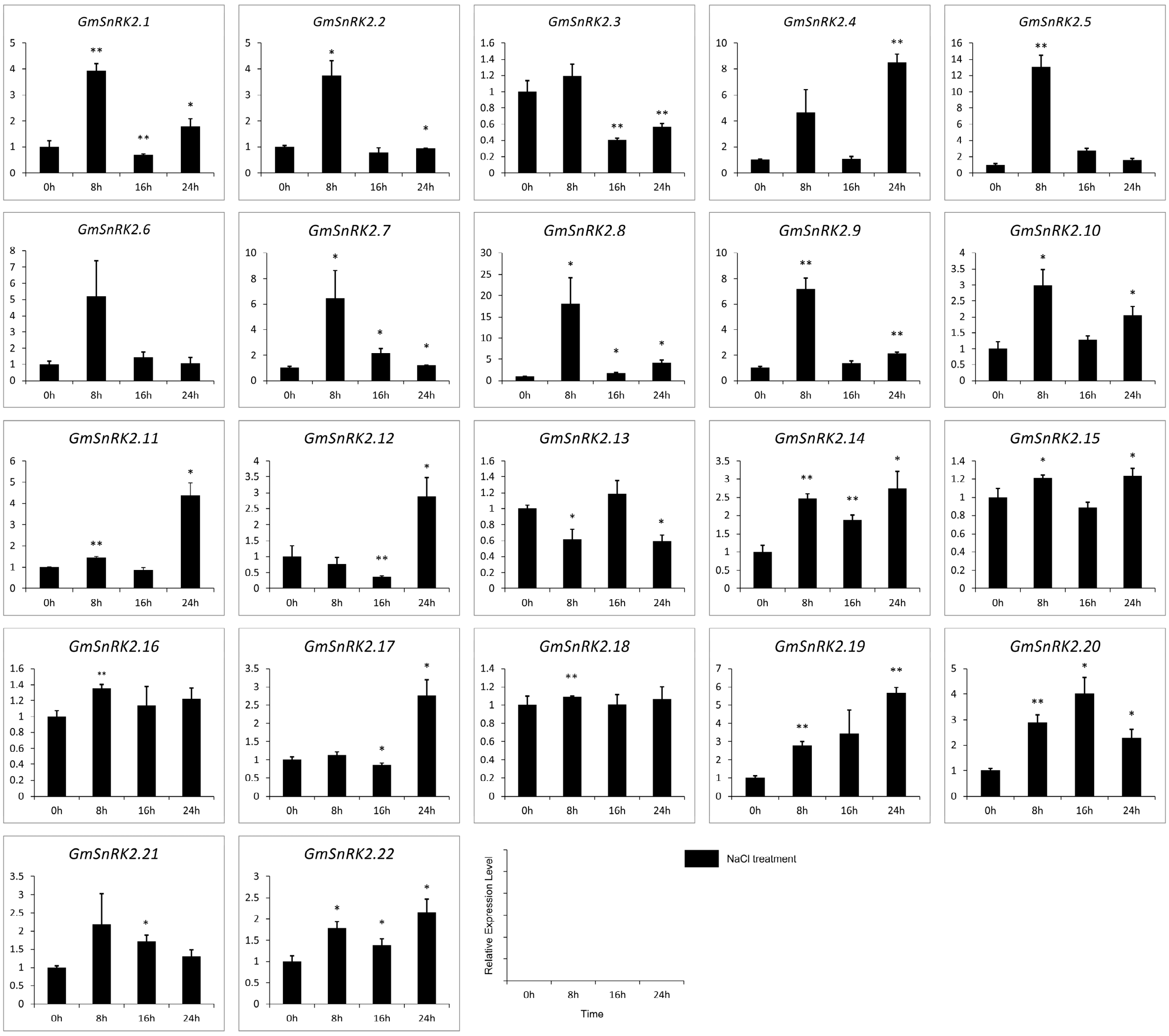

Figure S2. Expression patterns of the 22 *GmSnRK2s* under NaCl treatments with bar diagrams. The transcript levels of *GmSnRK2s* were normalized against *GmSKIP* transcript levels using $2^{-\triangle\triangle Ct}$. Error bars indicate the standard deviation. Statistically significant differences (Student's t–test) are indicated as follows: * $p < 0.05$, ** $p < 0.01$.

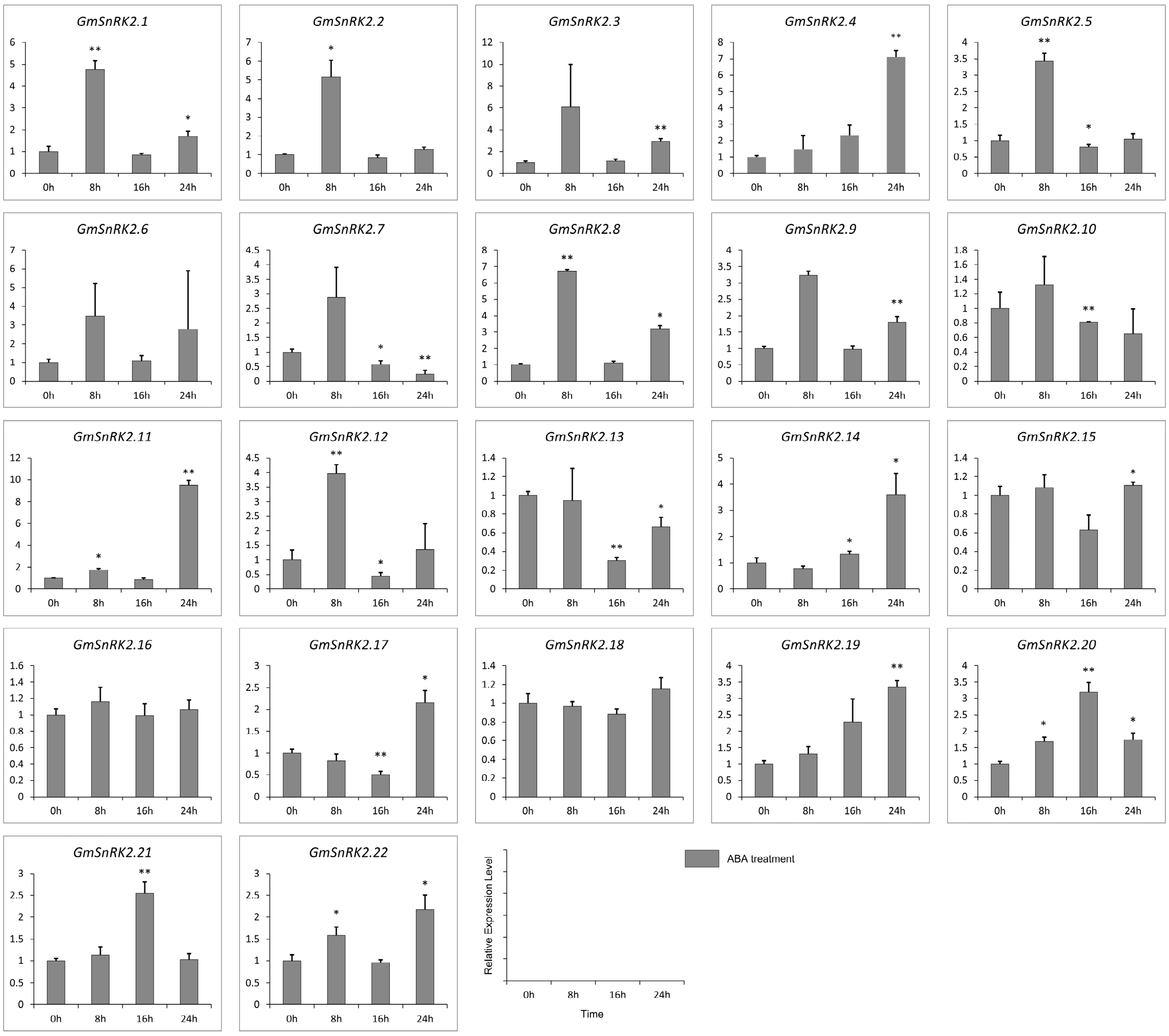

Figure S3. Expression patterns of the 22 *GmSnRK2s* under ABA treatments with bar diagrams. The transcript levels of *GmSnRK2s* were normalized against *GmSKIP* transcript levels using $2^{-\triangle\triangle Ct}$. Error bars indicate the standard deviation. Statistically significant differences (Student's *t*–test) are indicated as follows: * $p < 0.05$, ** $p < 0.01$.

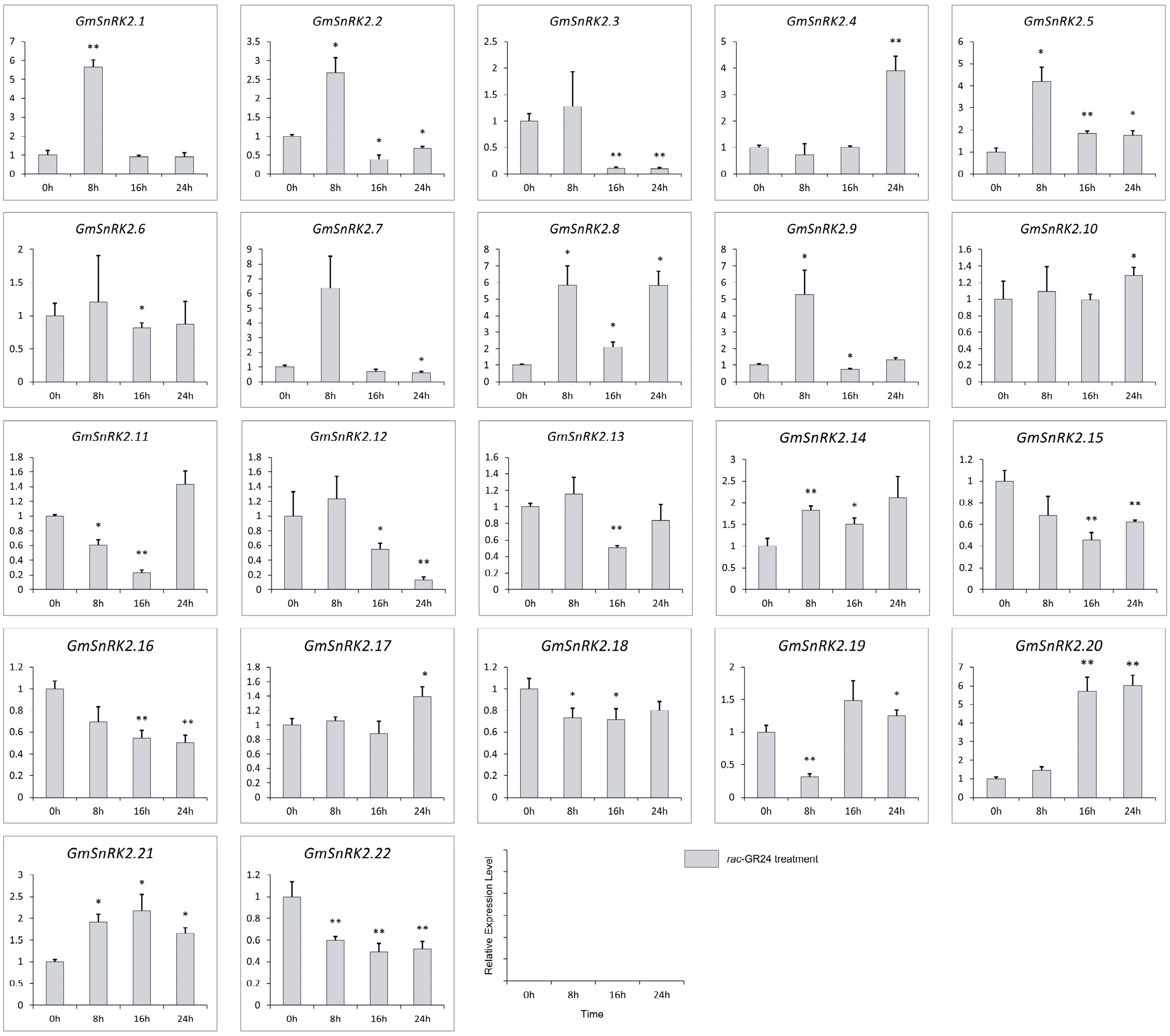

Figure S4. Expression patterns of the 22 *GmSnRK2s* under SLs treatments with bar diagrams. The transcript levels of *GmSnRK2s* were normalized against *GmSKIP* transcript levels using $2^{-\triangle\triangle Ct}$. Error bars indicate the standard deviation. Statistically significant differences (Student's *t*–test) are indicated as follows: * $p < 0.05$, ** $p < 0.01$.

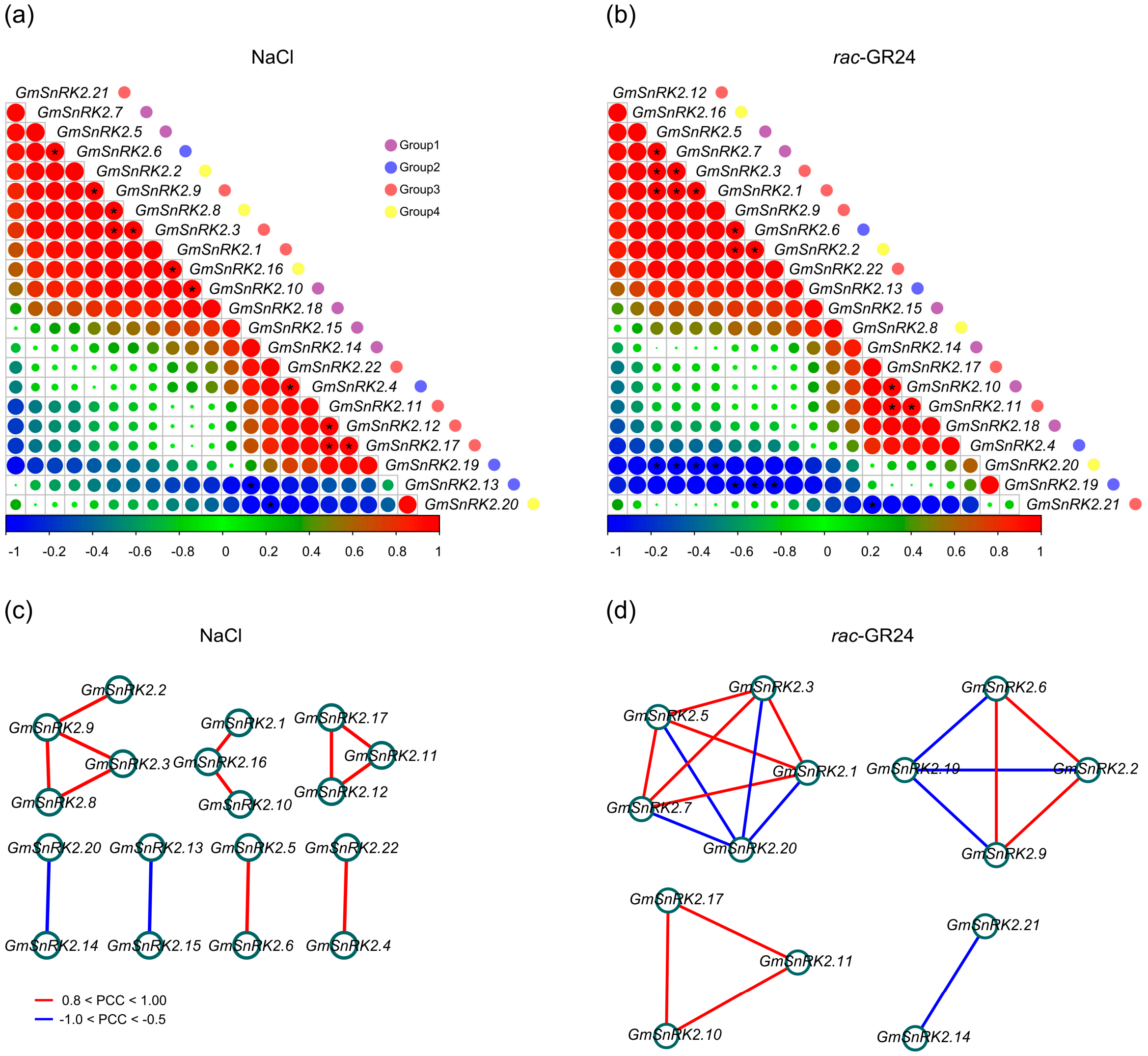

Figure S5. Correlations and co-regulatory networks of *GmSnRK2s* under NaCl and SLs treatments. Correlation analysis of *GmSnRK2* genes under NaCl (**a**) and SLs (**b**) treatments were performed based on the PCCs of gene pairs calculated using R package program. Correlations were indicated by the size and color of circles. Below bar represented the correlation values of PCCs. Red and blue indicate positive correlation and negative correlation, respectively. Gene name was listed at the right. Group1, Group2, Group3 and Group4 were marked as purple, blue, red and yellow circles, respectively. Black star represented the correlation with *p*−value ≤ 0.05. Co-regulatory network of *GmSnRK2s* under NaCl (**c**) and SLs (**d**) treatments were illustrated by Cytoscape. The significant PCCs of gene pairs (*p*-value ≤ 0.05) were included, and the different correlation levels of gene pairs were marked by edge line with different colors as showed at the bottom.
